# Correction of Population Stratification in Large Multi-Ethnic Association Studies

**DOI:** 10.1371/journal.pone.0001382

**Published:** 2008-01-02

**Authors:** David Serre, Alexandre Montpetit, Guillaume Paré, James C. Engert, Salim Yusuf, Bernard Keavney, Thomas J. Hudson, Sonia Anand

**Affiliations:** 1 Genome Quebec Innovation Centre, McGill University, Montreal, Quebec, Canada; 2 Department of Medicine, McGill University, Montreal, Quebec, Canada; 3 Department of Human Genetics, McGill University, Montreal, Quebec, Canada; 4 Department of Medicine, McMaster University, Hamilton, Ontario, Canada; 5 Institute of Human Genetics, Newcastle upon Tyne, United Kingdom; 6 Ontario Institute for Cancer Research, Toronto, Ontario, Canada; Centre National de la Recherche Scientifique, France

## Abstract

**Background:**

The vast majority of genetic risk factors for complex diseases have, taken individually, a small effect on the end phenotype. Population-based association studies therefore need very large sample sizes to detect significant differences between affected and non-affected individuals. Including thousands of affected individuals in a study requires recruitment in numerous centers, possibly from different geographic regions. Unfortunately such a recruitment strategy is likely to complicate the study design and to generate concerns regarding population stratification.

**Methodology/Principal Findings:**

We analyzed 9,751 individuals representing three main ethnic groups - Europeans, Arabs and South Asians - that had been enrolled from 154 centers involving 52 countries for a global case/control study of acute myocardial infarction. All individuals were genotyped at 103 candidate genes using 1,536 SNPs selected with a tagging strategy that captures most of the genetic diversity in different populations. We show that relying solely on self-reported ethnicity is not sufficient to exclude population stratification and we present additional methods to identify and correct for stratification.

**Conclusions/Significance:**

Our results highlight the importance of carefully addressing population stratification and of carefully “cleaning” the sample prior to analyses to obtain stronger signals of association and to avoid spurious results.

## Introduction

Complex diseases result from the intricate interactions of multiple environmental and genetic factors. In most cases, common genetic risk factors explain, individually, only a small proportion of the variance of quantitative traits and show modest associations between affected and non-affected individuals. Currently, most association studies include several hundred cases and controls from one single population, but the sample sizes are out of necessity increasing as a result of the expected relatively modest associations. In addition, the recent release of detailed descriptions of genetic diversity in non-European populations, such as those provided by the International HapMap project [1], will shift the focus from mostly Caucasian-centered studies to diverse populations from various geographic origins. For example, GlaxoSmithKline recently started an initiative to generate and publicly release large-scale genotype information from samples collected around the world [2]. This is appropriate since the majority of the global health burden is in low and middle income countries that include many individuals of non-European origins. Therefore studies are needed to examine the association of genetic markers for various diseases in multiple ethnic groups. Another trend affecting the recruitment strategy of genetic/epidemiologic studies is the collection of biological materials (i.e. blood and DNA) from a very large number of individuals (i.e. several hundreds of thousands) regardless of their health status. These prospective cohort studies will later allow designing nested case/control studies for any disease that is relatively common in the population [3], [4]. All these changes in recruitment strategies will require the development of specific methods for analyzing multi-ethnic and/or multi-center samples. Here, we describe practical methods for adequately designing and conducting population-based association studies with multi-center recruitment in which a large number of markers are genotyped. We use as an example more than 9,000 individuals (about half of whom are cases of first acute myocardial infarction and half are matched controls) from three ethnic groups recruited from 154 centers in the INTERHEART study and genotyped at 1,536 SNPs in 103 candidate genes. We describe an approach to efficiently select a set of tagging SNPs that captures most of the genetic diversity in populations with different allele frequencies and linkage disequilibrium patterns, and present several methods to efficiently identify and correct possible problems arising from population stratification and relatedness among subjects.

## Materials and Methods

### Samples

We analyzed individuals recruited for the INTERHEART study [5], a global case/control study of risk factors for acute myocardial infarction (MI) involving 29,972 individuals recruited from 262 centers in 52 countries. Informed written consent to obtain the baseline information and to collect and store the genetic and other biologic specimens was obtained from 21,508 individuals (including all individuals analyzed in this study). INTERHEART was approved by appropriate regulatory and ethics committees in all participating countries and centers and by the Institutional Review Board of McGill University Faculty of Medicine. To identify incident cases of acute MI, all patients, irrespective of age, admitted to the coronary care unit (or an equivalent cardiology ward) within 24 hours of symptom onset were screened. Cases were eligible if they had characteristic symptoms plus electrocardiogram changes indicative of a new MI (new pathologic Q waves, at least 1 mm ST elevation in any 2 or more contiguous limb leads or a new left bundle branch block, or new persistent ST-T wave changes diagnostic of a non-Q wave MI) or a plasma level of cardiac troponin level above that considered normal in the hospital/institution where the patient was registered. For each case, at least one control of the same age (±5 years) and sex was recruited from the same centre. Controls were defined as individuals who had no previous diagnosis of heart disease or history of exertional chest pain. Eligible controls were classified as i) hospital-based, defined as patients attending the hospital or outpatient clinics for the following reasons: refraction and cataracts, physical check-up, routine pap smear, routine breast exam, elective minor surgery for conditions that were not obviously related to CHD or its risk factors, elective orthopedic surgery (eligibility dependent on ability to complete physical measures), or ii. patients attending the hospital or outpatient clinics for: outpatient fractures, arthritic complaints, plastic surgery, hemorrhoids, hernias, hydroceles, routine colon cancer screening, endoscopy, minor dermatologic disorders; or ii) community-based, defined as visitors or relatives of a patient from a non-cardiac ward, or an unrelated (not first-degree relative) visitor of a cardiac patient. 58% of controls in INTERHEART were hospital-based and 36% of controls were community-based, and results were similar with both types of controls. In the remainder of the controls, 3% were from an undocumented source, and 3% were recruited through the WHO MONICA study in Göteborg, Sweden. Exclusion criteria for controls were identical to those described for cases. Structured questionnaires were administered to all cases and controls to obtain information on demographic factors (including self-reported ethnicity) as well as socioeconomic and health status. Non-fasting blood samples (20 mL) were drawn within 24 hours of hospital admission from each individual and centrifuged. These were separated into 6 aliquots (2 serum, 2 plasma, 1 citrate and 1 buffy coat) and frozen immediately at −20°C or −70°C after processing. Samples were shipped by courier to the National Blood Storage Site where they are stored in liquid nitrogen (−196°C). Finally, nitrogen vapor tanks were shipped to the Core Laboratory at the Population Health Research Institute (PHRI), Hamilton Canada for central long term storage. Samples collected among Chinese had to remain in China for legal reasons, and were shipped to the core lab in Beijing at the Fu Wai Hospital. We extracted DNA from blood samples using the Gentra Autopure LS isolation system (Gentra Systems Inc, Minneapolis, USA) according to the manufacturer's instructions. For this project, we analyzed 8,975 individuals with self-reported ethnicity defined as “Arab”, “South Asian” or “European” regardless of their geographic locations as well as 316 individuals from Nepal and 460 individuals from Iran who self-reported their ethnicity as “Other Asian”. Table 1 shows the countries in which the individuals genotyped have been recruited (see also Supplemental Figure S1). Following the approach used in the original INTERHEART analysis of nine modifiable risk factors and acute MI, we initially grouped people recruited from Nepal who reported their ethnicity as “other Asian” together with South Asian individuals and people recruited from Iran who reported their ethnicity as “other Asian” with Arabs. For sake of simplicity, we will refer to these three datasets as the European, South Asian and Arab population samples throughout the manuscript.

**Table 1:** Origin of the individuals used in the study.

	*Arab*	*European*	*South Asian*	*Other Asian*
Argentina		100		
Australia		433	5	
Bahrain	45		21	
Bangladesh			414	
Botswana		13	3	
Brazil		44		
Canada		109	2	
Chile		4		
Colombia		2		
Croatia		481		
Egypt	1037	1		
Hungary		152		
India			358	
Iran				460
Italy	1	303		
Japan		2		
Kenya			1	
Kuwait	669			
Malaysia		1	58	
Mozambique		4	12	
Nepal				316
Pakistan		1	966	
Philippines		2		
Poland		1301		
Qatar	20	1	56	
Russia		539		
Singapore		1	46	
South Africa		5	58	
Spain		141		
Sri Lanka			190	
Sult. Oman	241			
Sweden	3	571	1	
Thailand			2	
U.S.A.		53		
UAE	83	15	387	
Zimbabwe		13	4	

In addition, we genotyped the same SNPs in 1,062 individuals from the HGDP-CEPH Human Genome Diversity Cell Line Panel [6] later referred to as HGDP-CEPH panel. These individuals come from 52 populations representing most of the inhabited geographic areas of the world.

### Gene selection

Candidate genes were selected according to previous reports of association with MI or with one of the nine modifiable risk factors associated with MI [5], with a particular emphasis on lipid metabolism (see [7] for details).

### SNP selection

We retrieved the chromosome coordinates of each selected gene according to its refSeq annotation and included 10 kb of upstream and downstream DNA sequence to capture possible *cis*-regulatory variants. Overlapping gene regions (such as the APOA1-APOA4-APOC3 gene cluster) were concatenated into a single locus. We then retrieved the genotypes for all SNPs genotyped in these regions by the International HapMap project [1] (release 16) for all unrelated individuals from the following populations: individuals from Utah, USA, with northern and western European ancestry (CEU), individuals from the Yoruba people in Ibadan, Nigeria (YRI) and Han Chinese from Beijing, China (CHB). We used LD-select [8] separately for each region (i.e. gene or locus containing several genes) and each population (CEU, CHB and YRI) and identified possible tagging SNPs using a linkage disequilibrium (LD) cut-off of r^2^>0.8 and a minor allele frequency (MAF) of 5%. Finally, based on these results we selected the minimal set of tagging SNPs such that, in each population, every SNP (with MAF>5%) is either directly genotyped or in LD (r^2^>0.8) with one of the genotyped SNPs (see Supplemental Text S1 on-line).

In addition, we included all coding non-synonymous SNPs with a MAF larger than 5% (109 cSNPs, including 54 non-tSNPs) as well as SNPs that have been shown in the literature to be directly associated with MI, lipid metabolism or one of the other intermediate phenotypes relevant for the study of MI (145 SNPs, including 81 non-tSNPs). The final list of SNPs genotyped is shown in Supplemental Table S1.

### Genotyping

1,536 SNPs were genotyped using Illumina's GoldenGate technology based on allele-specific primer extension followed by highly multiplex PCR using universal primers [9]. 1,453 SNPs were successfully genotyped in more than 95% of the individuals of each population sample and are analyzed here (Supplemental Table S1). Individuals genotyped at less than 95% of the SNPs and those with genotypes at markers located on the sex chromosomes incompatible with their reported sex were excluded from further analyses (N = 387, see Supplemental Table S2 for a detailed breakdown).

### Estimation of relatedness

For each SNP, we determined whether two individuals from the same population sample shared 0, 1 or 2 allele(s) and averaged the allele sharing over all genotyped SNPs. We then compared the proportion of shared alleles for every pair-wise comparison within one population sample to a normal distribution and displayed the results in a quantile-quantile (QQ) plot.

After excluding identical, or nearly identical, samples (i.e., more than 99% of alleles shared, N = 170), we randomly selected 88 individuals from pairs that shared more than 83% of their alleles. This value corresponds to the relatedness cut-off empirically estimated (see Results for details). We successfully genotyped 87 of the individuals at 99 microsatellite loci. We performed a kinship analysis using the ML-relate program [10] that uses a Bayesian approach to estimate relationship between pairs of individuals.

To detect whether cases were significantly more related to each other than the controls to each other (or inversely), we tested in each population sample the distribution of allele sharing among cases to the distribution of allele sharing among controls. We calculated all pair-wise comparisons of allele sharing between two cases and all pair-wise allele sharing between two controls and tested the difference of the means of the two distributions by a Welch Two Sample t-test. We assessed the significance of the t-statistic by 300 permutations: for each population sample, we randomly assigned the individuals into two groups (i.e. regardless of the disease status) and tested the difference between the mean of the two distributions consisting of all possible pair-wise comparisons within each group. To evaluate the power of these analyses, we used unrelated individuals from the Saguenay-Lac St-Jean region (SLSJ, Quebec, Canada) that have been genotyped at the same SNPs [7]. We calculated every pair-wise comparison of two individuals from this population and tested this distribution against all pair-wise comparisons of two European controls from the INTERHEART study. We controlled for possible population differentiation by testing the distribution of pair-wise comparisons between one individual from the SLSJ region and one INTERHEART European individual against pair-wise comparisons of the Europeans controls.

### Assessment of population stratification

To estimate population stratification at a gross level, we used the program STRUCTURE [11]. This program uses a Bayesian approach to assign individuals into a pre-specified number (*K*) of “populations” according to their genotypes. These populations are determined such that linkage disequilibrium among unlinked markers and deviations from Hardy-Weinberg are minimized in each of them. We allowed the individuals to be admixed from two or more populations and used a model of correlated allele frequencies which yields stronger clustering [12]. Every analysis was replicated thrice and consisted of 200,000 burn-in steps followed by 200,000 Markov Chain Monte Carlo steps. We selected genotypes from SNPs distant from at least 50,000 bp to decrease the chance that they are in LD with each other. Two sets of SNPs were generated: a first set of 133 SNPs randomly selected according to our distance criteria, and a second set composed of the 127 SNPs highly differentiated across populations (based on Fst estimates calculated after grouping the HDGP-CEPH individuals by continents, [13]. This second set of SNPs led to higher discrimination power (Supplemental Figure S2) and only results obtained with this set are presented in further analyses. The analyses with STRUCTURE were performed i) separately on each population sample after addition of all individuals from the HGDP-CEPH panel (except for Native American and Oceanian individuals since prior studies of these populations have emphasized the importance of genetic drift leading to large differences in allele frequencies) or ii) on the entire dataset combining all European, Arab and South-Asian INTERHEART individuals.

### Second generation population samples

We generated second generation population samples by first removing problematic samples and centers: 1) we randomly excluded one individual from each pair of related individuals (N = 131), 2) all individuals that were clustered by STRUCTURE among sub-Saharan Africans or East Asians (N = 104), and 3) all individuals from two centers that showed a very high proportion of problematic samples (including more than 10% discrepancies between reported and genetically-inferred sex, N = 719). In addition, all Nepalese and Iranian individuals were removed from, respectively, the South Asian and the Arab population sample (N = 776). Supplemental Table S2 shows the detailed breakdown per population sample.

### Associations between genotypes and Apolipoprotein B concentrations

We tested, separately in each population sample, the association between genotypes and ApoB levels in blood for each SNP by an analysis of variance (ANOVA). We used sex, age and waist circumference as covariates in these analyses and excluded individuals with diabetes (defined as self-reported diabetes, on medication pre-admission for diabetes, oral hypoglycemics, insulin or with HbA1c>7%) or on pre-admission medication for lowering cholesterol or blood pressure (inclusion of diabetic individuals led to the same strong associations with ApoB, data not shown). We also included as covariates for some of the analyses the recruitment center and the coefficients of ancestry inferred by STRUCTURE for each individual (using the results obtained by analyzing all individuals from the three population samples together). To estimate whether multiple significant associations from the same region were independent or simply due to LD, we tested hierarchically the associations by successively including the genotypes of stronger associations as covariates.

## Results

### Identification of related individuals

To estimate whether the datasets made of individuals of a same self-reported ethnicity were roughly genetically homogenous, we calculated in each population sample the proportion of shared alleles between every pair of individuals. If individuals are sampled randomly from a homogeneous random-mating population, we expect every individual to be, on average, equally distant genetically from everybody else (since information from many unlinked loci is summarized). We thus plotted the distribution of allele sharing for all pair-wise comparisons within each population sample against a normal distribution (see Figure 1 for the European individuals and Supplemental Figure S3 for the other two datasets). Overall, the distributions appear roughly normal (i.e., we obtain a straight line on the QQ-plot for most of the range) but with significant deviations on both extremes. We observed a dramatic deviation on the right-hand side of the graph for the pairs of individuals with a proportion of allele sharing larger than 0.83 that could indicate sampling of related individuals. The most extreme case in the European sample consists of identical or nearly identical (>99%) genotypes obtained from 16 pairs of supposedly different individuals. The great majority of the pairs with a high proportion of shared alleles (i.e. larger than 0.83) are composed of individuals recruited in the same center. Overall we identified 71 likely related individuals (39 pairs) in the European population sample, 75 (41 pairs) in the South Asian sample and 97 (60 pairs) in the Arab sample. To test whether these individuals were actually related, we randomly selected 87 individuals from pairs with a very high proportion of allele sharing (>0.83), after exclusion of identical or nearly identical DNAs (>0.99), and genotyped them at 99 microsatellite loci. Kinship analyses using the Bayesian approach implemented in ML-relate [10] identified the same pairs of related individuals, with different degrees of relatedness: 71 pairs of parent/offspring, 28 full-siblings and 12 half-siblings.

**Figure 1:**
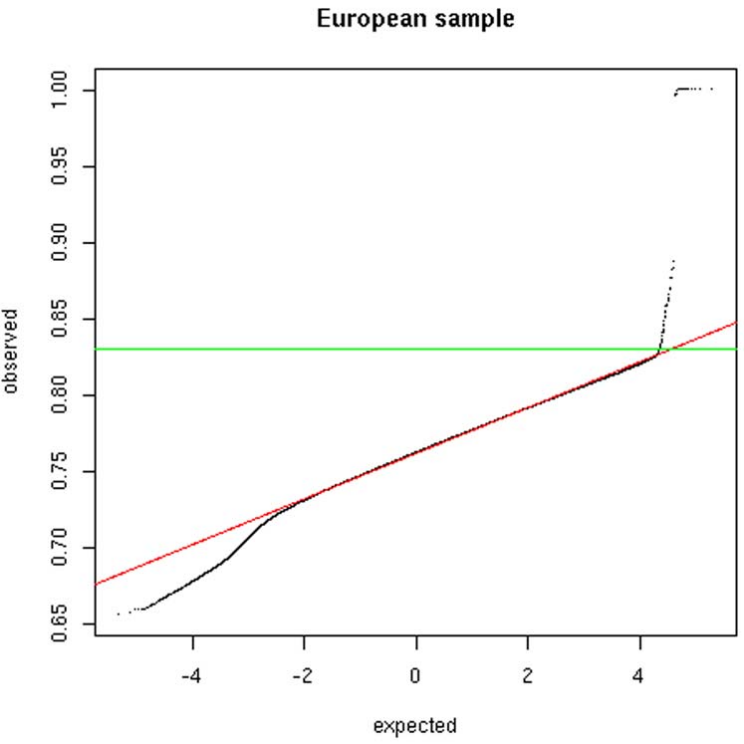
Distribution of pair-wise allele sharing among the INTERHEART European individuals. The graph shows the QQ plot of the distribution of all pair-wise measures of allele sharing against a normal distribution (the red line displays the expectation). The green line shows to the empirical cut-off used to identify related individuals (correspond to an allele sharing larger than 83%). The deviation on the left-hand side of the graph (i.e. low allele sharing) corresponds to pairs of individuals originating from different sub-populations.

The presence of closely related individuals can generate spurious results but is unlikely to strongly influence association studies unless they make up a large proportion of the dataset. On the other hand, the possibility that the cases are, on average, more closely related to each others than are the controls (or inversely) is particularly worrying since this difference in genealogy depth could potentially generate large numbers of false positives [14], [15]. We examined this possibility in our study by testing, in each population sample, the distribution of pair-wise allele sharing among cases against the distribution obtained by pair-wise comparisons among controls. We assessed the significance of the t-statistic obtained by 300 permutations (see Supplemental Figure S4 for the Europeans). The difference in allele sharing between cases and controls was not significant in any population sample (p = 0.75 for the European individuals, p = 0.77 in South Asians and p = 0.75 in Arabs). We evaluated the power of these analyses by estimating the distribution of allele sharing among unrelated individuals recruited from a founder population of the Saguenay Lac S^t^-Jean region of Quebec, Canada. For these individuals, we observed a significant increase in allele sharing compared to the European individuals from the INTERHEART study (p<0.005). We validated that this difference resulted from higher average relatedness and not from population differentiation by comparing pair-wise allele sharing between one individual from the SLSJ region and one European from the INTERHEART study and testing this distribution against the within-European distribution of allele sharing (p = 0.16). Non-parametric testing (Two-sample Kolmogorov-Smirnov) yielded, qualitatively, similar results (data not shown).

### Analysis of gross population stratification

Figure 1 also shows an excess of pairs on the left-hand side of the graph relative to a normal expectation. These pairs of individuals are more different genetically (i.e. less allele shared) than the vast majority of the pairs and this could indicate population stratification in the sample or the presence of individuals with an incorrect self-reported ethnicity. As a first attempt to identify individuals genetically different from the rest of the samples, we analyzed each population sample separately using the program STRUCTURE [11]. STRUCTURE is a Bayesian algorithm that uses genotype information from all individuals without considering their origins and assigns them into a chosen number of populations (see Materials and Methods for details). Since this algorithm relies on the estimation of allele frequencies in different populations, it is easier to identify groups of individuals than a few outliers in a relatively homogenous population [11], [12]. We thus spiked each population sample before analysis with 914 individuals from the HGDP-CEPH panel [6] originating from several geographic locations in Africa, Europe and Asia (see Materials and Methods). These individuals also enable us to estimate the discrimination power of our analysis and the level of population differentiation that can be identified. Using 127 SNPs highly differentiated among populations from different continents (see Materials and Methods), we were able to pin-point a few individuals whose genotypes were more compatible with an ancestry from Sub-Saharan Africa or South-East Asia than their “European”, “Arab” or “South-Asian” self-reported origin (Supplemental Table S3). Interestingly, in many of these cases, the ancestry inferred from the genotypes best fitted the individual's geographic origin than his/her self-reported ethnicity. For example, three individuals from Zimbabwe self-described as Europeans displayed very high coefficient of ancestry from the African population (i.e. larger than 90%). In addition, a large proportion of the individuals recruited from Nepalese centers cluster with South-East Asian individuals from the HGDP-CEPH (Supplemental Table S3). All Nepalese individuals self-reported their ethnicity as “Other Asian” but were analyzed together with “South Asians” based on similarity in cultural practices in previous publications of the INTERHEART study [5], [16], [17]. The analysis of each population sample separately lacked power to separate individuals from Europe, the Middle East and South Asia as indicated by the assignments of the HGDP-CEPH individuals (i.e., we did not identify any clustering of the HGDP-CEPH individuals at the sub-continental level). We thus reanalyzed with STRUCTURE all INTERHEART individuals pooled together (after exclusion of the few outliers with Sub-Saharan African or East Asian ancestry). Figure 2 shows the results of this analysis using K = 3 populations. With enough individuals from each group, STRUCTURE is able to better estimate the allele frequencies corresponding to the three main self-reported ethnicities (i.e. Europeans, South Asians and Arabs) and consequently, assigns more than 90% of the individuals in the population corresponding to their self-reported ethnicity with a coefficient of ancestry larger than 0.85 (see Figure 2 and Supplemental Figure S2). The remaining individuals could represent random fluctuations due to our limited power (only 127 SNPs were used in this analysis) or differences between genetically-inferred and self-reported ethnicity). We did not detect any clear correlation between the assignment coefficients estimated by STRUCTURE and, either the geographic origin of the samples or their case/control status. However, we observed that a large proportion of the individuals recruited in Iranian centers that self-described their ethnicity as ‘Other-Asian’ (but were gathered in the Arab population sample in this study) were assigned among “Europeans”.

**Figure 2:**
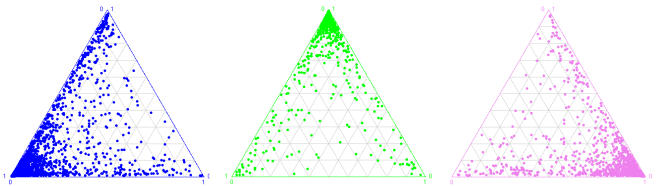
Genetic clustering of the INTERHEART individuals inferred by STRUCTURE. “European” (blue dots), “Arabs” (green dots) and “South Asian” (pink dots) individuals are displayed according to their coefficients of ancestry in three populations (K = 3) as estimated by STRUCTURE using 127 SNPs. The coefficients of ancestry display separately for each population samples were inferred from a single analysis (i.e. all individuals combined) and are represented using the same axes. See also Supplemental Figure S2 for the distribution of the coefficients of ancestry.

Based on the results of these analyses we generated second generation datasets after exclusion of problematic samples. We randomly excluded one individual from each pair of related individuals, all individuals that were clustered by STRUCTURE among sub-Saharan Africans or East Asians and all Nepalese and Iranian individuals. In addition, we excluded all individuals from two centers that showed a very high proportion of problematic samples (including more than 10% discrepancies between reported and genetically-inferred sex). This consequently reduced our sample sizes to 4,069 individuals in the European population sample (starting from 4,292), 2,450 in the South Asian sample (out of 2900, including 316 Nepalese) and 1,399 individuals in the Arab sample (out of 2559, including 460 Iranians).

### Correcting association analysis for possible residual stratification

We tested separately in each population sample the association between genotypes and Apolipoprotein B (ApoB) concentration (see Materials and Methods for details). Figure 3 shows the distribution of the p-value obtained for each SNP in the South-Asian dataset. The figure shows a global deviation (towards more significant associations) from the pattern expected by chance if there is no association between genotypes and ApoB concentration. This deviation is not limited to a few outliers but affects the entire distribution. This could be an indication that many SNPs (i.e. several hundred) in our panel are significantly associated with ApoB level or, alternatively, that a previously undetected stratification in the dataset affects the results. We first tried to correct this global deviation by using the coefficients of ancestry estimated by STRUCTURE for each individual as covariates in the ANOVA. This did not lead to any significant difference in the distribution of the p-values (see Supplemental Figure S5). We then tested whether the geographic origin of the individuals could influence the associations. After using the recruitment centers as covariates of the analyses, the distribution of the p-values for the South-Asian individuals fitted much better the distribution expected under no association, and only five SNPs (most notably rs429358 in APOE) showed significant deviation from the expectation and strong association with ApoB concentration (Figure 3). Similar patterns were observed in the Arab and, to a lesser extent, in the European datasets (data not shown). Correcting the association tests for the recruitment centers thus led to a dramatic change in the overall distribution of the associations with some of the SNPs showing up to two orders of magnitude decrease in statistical significance. It is important to note here that the stratification observed among centers is not due to a systematic difference in DNA preparation or storage between centers. The INTERHEART protocol requires that, for every case recruited, at least one control (same sex, same age) is recruited from the same center. Blood samples (or buffy coats) from cases and controls are then shipped to Canada and treated identically (after randomization). However, due to stochastic failures at different stages (e.g. DNA extractions, genotyping) some centers included more cases than controls (or inversely) at the end of the study which contributes to the observed stratification effect (in combination with allele frequency differences among centers).

**Figure 3:**
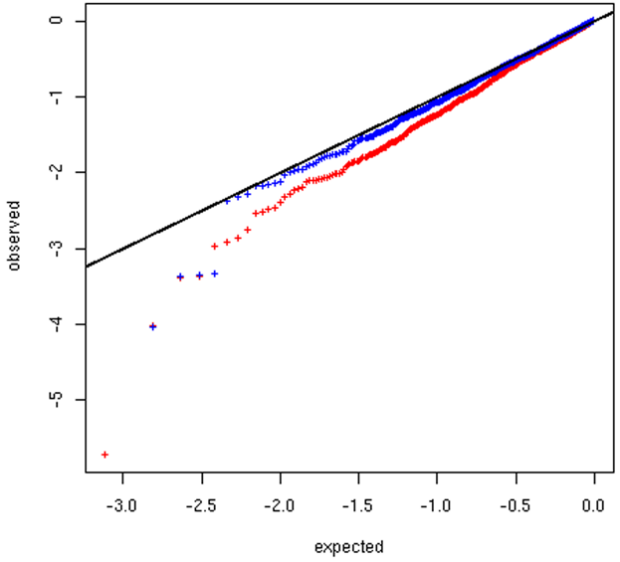
Distribution of the p-values of the associations between genotypes at 1,453 SNPs and ApoB level in South-Asians. The plot shows the observed distribution of the p-values (y-axis) against the expectation under a model without any association (grey crosses and x-axis). The axes are in logarithmic scales. Red crosses correspond to the association between ApoB and the genotypes at one SNP without any correction. Blue crosses stand for the same tests using recruitment centers used as additional covariates.

In the European dataset (but not in the South-Asian and Arab datasets), the distribution of p-values shows a bump with a higher significance level for the SNPs with p<0.05 (74 SNPs) than we would expect by chance (Supplemental Figure S6). Interestingly, most of the strongest associations come from SNPs located in a few genes. We thus tested whether this deviation was due to the carry-over of a limited number of signals to many SNPs in strong LD with each others. We reanalyzed the associations between the genotypes and ApoB level conditional on the genotypes of SNPs with the strongest associations (see Materials and Methods). After correcting for the signal of the five strongest associations, the entire distribution becomes indistinguishable from the expected distribution (Supplemental Figure S6).

### Effect of cleaning the dataset

To estimate the influence of stratification on the results obtained and the loss of power resulting from the reduction in sample size, we contrasted the results of the associations with ApoB concentration prior to and after “cleaning” in each dataset. In all population samples, we observe a reduction in the deviation of the p-value distribution from the expectation after removing outlier individuals and/or centers (i.e. in the second generation population samples). The changes are more dramatic in the Arab dataset than in the South-Asian and European datasets (Supplementary Figure S7). The effect of cleaning the datasets does not affect evenly all markers and some of the associations between genotypes and ApoB concentration changed more dramatically than others. Consequently, the markers most strongly associated with ApoB concentration differ (Table 2). Interestingly, we note that after cleaning, the SNP most strongly associated with ApoB level in all three population samples (rs429358 in APOE) replicates well supported associations [18], [19]. In addition, the p-values for the most significant associations are only moderately changed (decreased or actually improved) despite the loss of 3.6 to 40% of the samples (Table 2).

**Table 2:** Five strongest associations with ApoB level in each population sample using raw data and second generation datasets.

SOUTH ASIAN											
Raw data (N = 2065)						Second generation population sample* (N = 1836)					
w/o Centers			w/Centers			w/o Centers			w/Centers		
rs429358	2.03E-07	APOE	rs429358	4.77E-08	APOE	rs429358	1.90E-06	APOE	rs429358	6.75E-07	APOE
rs6511720	2.92E-04	LDLR	rs6511720	1.12E-04	LDLR	rs6511720	9.52E-05	LDLR	rs6511720	8.93E-05	LDLR
rs1713223	5.36E-04	APOB	rs9650662	1.23E-03	FDFT1∧	rs1534863	4.04E-04	FDFT1∧	rs4762	4.22E-04	AGT
rs1534863	1.20E-03	FDFT1∧	rs1534863	1.35E-03	FDFT1∧	rs9650662	4.29E-04	FDFT1∧	rs1534863	4.47E-04	FDFT1∧
rs9650662	1.25E-03	FDFT1∧	rs4762	2.67E-03	AGT	rs4762	1.05E-03	AGT	rs9650662	4.55E-04	FDFT1∧

ARAB											
Raw data (N = 1527)						Second generation population sample* (N = 870)					
w/o Centers			w/Centers			w/o Centers			w/Centers		
rs7449650	2.07E-06	LPA	rs429358	7.37E-06	APOE	rs1328757	6.72E-05	PCK1	rs429358	3.26E-05	APOE
rs3931914	1.56E-05	HMGCR'''	rs3758294	1.24E-04	ABCA1	rs429358	6.96E-05	APOE	rs6511720	3.27E-04	LDLR
rs3758294	1.64E-05	ABCA1	rs5995472	5.23E-04	LGALS2	rs7449650	1.16E-04	LPA	rs1328757	5.34E-04	PCK1
rs429358	2.40E-05	APOE	rs3827608	1.12E-03	HMGCR'''	rs6511720	1.30E-04	LDLR	rs865716	5.87E-04	SCARB1
rs3827608	1.07E-04	HMGCR'''	rs2291427	2.05E-03	ALOX5	rs8102912	2.55E-04	LDLR	rs989892	6.97E-04	SCARB1

EUROPEAN											
Raw data (N = 2595)						Second generation population sample* (N = 2501)					
w/o Centers			w/Centers			w/o Centers			w/Centers		
rs429358	4.31E-10	APOE	rs429358	1.51E-09	APOE	rs429358	4.37E-10	APOE	rs429358	1.79E-09	APOE
rs693	4.76E-05	APOB	rs662799	2.66E-05	APOA5	rs693	9.50E-05	APOB	rs662799	4.71E-05	APOA5
rs2072560	3.10E-04	APOA5	rs2072560	3.33E-05	APOA5	rs2072560	1.65E-04	APOA5	rs2072560	5.66E-05	APOA5
rs662799	5.40E-04	APOA5	rs651821	5.49E-05	APOA5	rs662799	2.34E-04	APOA5	rs651821	6.90E-05	APOA5
rs7575840	6.69E-04	APOB	rs693	5.87E-05	APOB	rs651821	2.87E-04	APOA5	rs693	7.89E-05	APOB

* see Materials and Methods for details

∧ locus also contains CTSB

'' locus also contains COL4A3BP

## Discussion

### Detecting and correction for population substructure

One of the main drawbacks of population-based association studies (in comparison to family-based association studies) are their susceptibility to population stratification [20]–[23]. The presence of differing levels of relatedness among the samples or the existence of unnoticed sub-populations can induce both a loss of power in detecting true associations and generate spurious associations [14], [15], [22], [23]. These issues are likely to become even more crucial in the future since the effect of stratification increases with the sample size and since recruitment criteria are widening to obtain larger and multi-purpose cohorts. For example, UK Biobank, one of the largest on-going prospective studies, only excludes first-degree relatives and aims to obtain a global representation of the UK population including its ethnic minorities. We describe in our study a handful of simple methods that can be applied to any large scale genotyping projects (i.e. more than 1,000 SNPs) to identify and address possible stratification problems in the sample.

The INTERHEART study was originally designed as a “matched” case-control study but was unmatched in the analysis of nine modifiable risk factors [5] to minimize the loss of cases and controls for whom matching was not possible, given that there was general agreement for key results among the matched and unmatched data analyses. In this genetic analysis matching was not used since we often lacked genotypes of one of the two members of the matched pair (due to a failure in blood collection, DNA extraction or genotyping). As a consequence, the INTERHEART protocol, while specifically excluding the recruitment of related individuals as cases, authorized using a relative of one case as a control for another case. Numerous methods have been developed in the past to assess the degree of relationship among individuals (see [24], [25] for reviews). We showed here that, when enough markers are genotyped, a simple QQ-plot of allele sharing, as the one in Figure 1, allowed us to identify related individuals who almost always consisted of a case and one or more control individual(s). A more problematic issue arises if the cases are on average more related to each other than are the controls (or inversely): the global difference in the depth of the genealogies of each group can lead to differences in alleles frequencies and thus generate spurious associations. Since many of the individuals analyzed here were recruited from non-cosmopolitan areas, we were concerned that this could be an issue in the INTERHEART study. By comparing the mean pair-wise allele sharing observed among cases to that observed among controls, we showed that none of the differences observed was significantly larger than the difference observed by randomly assigning individuals into two groups. By contrast, the same analysis performed on unrelated individuals from a founder population of the Saguenay-Lac St-Jean from Quebec, Canada [26] revealed an overall shorter genealogy than the INTERHEART Europeans consistent with their demographic history. This result clearly indicates that our analysis has sufficient power to identify slight differences in relatedness and excludes differential average relatedness between cases and controls as a major issue in our population samples.

One of the most common arguments advanced to explain the lack of reproducibility in population-based association studies is the presence of undetected subpopulations in the sample, leading to spurious results (e.g. [27]). We expected this issue to be especially problematic in this study since the individuals (both cases and controls) were recruited in more than a hundred centers across the world and later grouped together based on their self-reported ethnicity. Several methods have been developed to address population stratification based either on correcting the test statistic to account for genetic heterogeneity in the sample, or on performing structured associations after the identification of subpopulations [20], [28]. Devlin and Roeder [14] proposed to use random SNPs as “genomic controls” to estimate the average effect of population substructure in the sample and then correct the test statistics accordingly. One limitation of this approach is that it assumes a constant effect of stratification or admixture over all loci and thus does not correct appropriately for markers located in regions of adaptive selection (i.e. loci where natural selection acted or is acting differently on different populations). This is a major drawback for whole genome scans: they include many SNPs in such regions that will not be sufficiently corrected (see [29] as an example). Even for candidate gene studies, this effect can critically hamper the association analyses since natural selection can greatly affect genetic diversity at disease genes [30]. In the INTERHEART study, several genes under investigation have been shown to differ drastically among populations due to the effect of natural selection [31]–[33]. We thus discarded using genomic controls (GC) to correct for stratification since GC selected randomly would not correct sufficiently for stratification in genes under selection, while selecting GC in genes under selection would inflate the correction coefficient and over-correct all other loci, resulting in a large loss of power. Instead, we opted to use the program STRUCTURE [11] that uses genotypes to group individuals according to their genetic ancestry. An alternative program, EIGENSTRAT [34], performs similar analyses but does not incorporate any defined genetic model and thus is not as efficient as Structure with only a few hundreds of independent markers (it is, on the other hand, computationally more interesting for genome-wide or other large dataset). We spiked our dataset with individuals of known ancestry genotyped at the same loci to better identify possible individuals with ancestry from Africa and South East Asia present in the INTERHEART dataset. This procedure, coupled with the use of highly differentiated SNPs (i.e. SNPs with high Fst) yielded better clustering and thus, a more powerful identification of outliers (see also [29]). Overall, the great majority of the individuals were gathered into the population corresponding to their self-reported ethnicity, consistent with previous reports showing high correspondence between self-reported ethnicity and genetic estimates of ancestry [35]. In addition, many individuals from Nepal, who all reported an “Other Asian” ethnicity but were grouped with “South Asian” individuals in previous analyses of the INTERHEART study, display high coefficients of ancestry from South East Asian populations as well as high heterogeneity in their assignments. This observation is consistent with previous reports of genetic heterogeneity in Tibeto-Burman populations [36], [37] and shows that the self-reported ethnicity correctly captured the genetic information but the later grouping of these individuals with South-Asian individuals lead to genetic heterogeneity. In contrast, we identified several clear outliers in each dataset, with in some cases the genetically inferred ancestry corresponding better to the geographic location than their self-reported ethnicities. These discrepancies could be due to clerical errors or sample mislabeling, or alternatively, represent true differences between self-reported ethnicity and genetic ancestry. In agreement with previous studies [38], we identified several Brazilian individuals self-described as “Europeans” that show high level of African ancestry which illustrates some of the limitations of using self-reported ethnicity. Unfortunately, we were underpowered to identify (or rule out) with STRUCTURE and the reduced number of markers available (∼130 selected SNPs) more subtle stratification levels due to intra-continental differences (e.g. [39]). The deviations in the distribution of the p-values observed in at least two datasets (i.e. South Asian and Arab) clearly indicate that the exclusion of the outliers identified by STRUCTURE was not sufficient to remove all stratification and illustrates that self-reported ethnicity on its own is not sufficient to protect against population stratification. However, we successfully corrected this overall inflation in the significance of the associations using the recruitment centers as covariates. This approach can be easily applied to other scenarios when, for example, the controls are recruited in different centers. In such cases, a simple test such as a QQ plot of the p-value distribution will indicate if the use of additional covariates is useful and if using the dataset is appropriate for drawing biological/medical conclusions.

Several studies have looked at the effect of stratification from a theoretical perspective and sometimes reached contradicting conclusions [22], [23], [40], [41] but few concrete examples have shown its influence on the results of a real association study [39], [42], [43]. Here we empirically show that cleaning-up the datasets to remove as much stratification as possible does influence the overall distribution of the association p-values. In particular, we demonstrate that even the strongest associations (i.e. the SNPs that are most likely to be reported as “significantly associated”) can differ according to the “state” of the dataset: while we observe significant differences among the results of each dataset prior to cleaning, the strongest association in all three cleaned datasets is due to one SNP in the ApoE gene (rs429358) known to be strongly involved in Apolipoprotein B concentration [19]. Interestingly, the loss of power resulting from a reduction in sample size (up to 40% in the Arab dataset) is almost completely compensated by the cleaner signal obtained: the strength of the confirmed associations is very similar or even improved in the cleaned datasets relative to the analysis performed with the raw data. This shows that cleaning up the datasets to obtain un-stratified samples, even at the cost of reduced sample size, is crucial to obtain reliable results. In our study, the genetic risk factors associated with the phenotype investigated seem to be similar in the different populations (e.g. APOE shows strong association with ApoB in all three population samples). In addition, the strongest signal comes from the presumably functional allele that has been directly genotyped. This represents the best case scenario to identify true associations in a stratified sample (even if the stratification will still generate spurious associations). If on the contrary, the risk factors associated with a particular trait differ among populations (e.g. if one would look at lactose tolerance, [44], or if the causative polymorphism is not directly genotyped and the LD patterns differ among populations, the power to detect true associations in a stratified population sample will be greatly decreased, resulting in both spurious associations and false negatives. This also illustrates a potential drawback of combining cohorts for different ethnicities in a single analysis: if the LD patterns surrounding the causative polymorphism(s) are different among populations or if the genetic risk factors are not shared across ethnicities, pooling individuals from diverse origin could lead to a loss of power (by diluting the effect observed at a given marker) instead of an increase due to the larger sample size.

## Supporting Information

Figure S1 Map showing the geographic origin of each INTERHEART individual analyzed in this study. Each pie graph shows if at least one individual with self-reported ethnicity defined as “European” (blue section), “South-Asian” (pink section) or “Arabs” (green section) has been recruited in the country (regardless of the number of individuals recruited, see Supplemental Table S1 for details). All individuals from Nepal and Iran reported their ethnicity as “Other Asian” and are displayed by a yellow section.
(0.50 MB TIF)

Figure S2 The graphs show the distribution of individuals according to their coefficients of ancestry from each population (K = 3). The left panel correspond to the assignments using 127 SNPs highly differentiated across population, the right panel to the assignments using 133 SNPs randomly selected.
(0.20 MB TIF)

Figure S3 QQ plot of the distribution of pair-wise allele sharing among the South Asian (left panel) and Arab (right panel) individuals against a normal distribution.
(0.06 MB TIF)

Figure S4 Estimation of cryptic relatedness in Europeans. The graph displays the distribution of the t-statistic obtained in 300 tests of the difference in means between the distributions of allele sharing within two groups of randomly assigned individuals (Welch Two Sample t-test). The red arrow shows the t-statistic obtained by testing the INTERHEART Europeans cases vs. controls. The green arrow corresponds to the comparison of the distribution of pair-wise allele sharing among the Saguenay Lac St-Jean (SLSJ) individuals vs. the allele sharing observed in Europeans from the INTERHEART study. The pink arrow shows the t-statistic obtained in the comparison of inter-sample allele sharing (i.e., one SLSJ individual compared to one European individual from INTERHEART) vs. the distribution of allele sharing in Europeans.
(0.07 MB TIF)

Figure S5 Effect of STRUCTURE on the distribution of the p-values for the associations between the genotypes and ApoB level in South-Asians. The plot shows the observed distribution of the p-values against the expectation under a model without any association (axes in logarithmic scales). Red crosses correspond to the association between ApoB and the genotypes at one SNP without any correction. Light blue crosses stand for the same tests using the coefficients of ancestry from STRUCTURE used as additional covariates
(0.08 MB TIF)

Figure S6 Distribution of the p-values for the associations between the genotypes and ApoB level in Europeans. Red crosses correspond to the non-corrected association between ApoB and the genotypes at one SNP. Blue crosses stands for the same tests after correcting for the signal of the five strongest associations (i.e. by conditioning the analyses on the genotypes at the five strongest associations).
(0.05 MB TIF)

Figure S7 Distribution of the p-values for the associations between the genotypes and ApoB level in raw and cleaned datasets. Crosses correspond to the association between ApoB and the genotypes at one SNP using the raw (x-axis) and the cleaned datasets (y-axis). Green, Pink and Blue crosses stand for respectively the tests in the Arab, South-Asian and European datasets.
(0.10 MB TIF)

Table S1 Description of the SNPs included in this study.
(0.04 MB PDF)

Table S2 Excluded samples
(0.01 MB PDF)

Table S3 Outliers identified by STRUCTURE with substantial ancestry from South-East Asia or Sub-Saharan Africa
(0.01 MB PDF)

Text S1 Tagging Efficiency
(0.42 MB DOC)
