# Supplementary figures and images for: Correction of Population Stratification in Large Multi-Ethnic Association Studies
Source: PLoS One. 2008 Jan 2;3(1):e1382. doi: 10.1371/journal.pone.0001382 (PMC2198793; doi:10.1371/journal.pone.0001382)

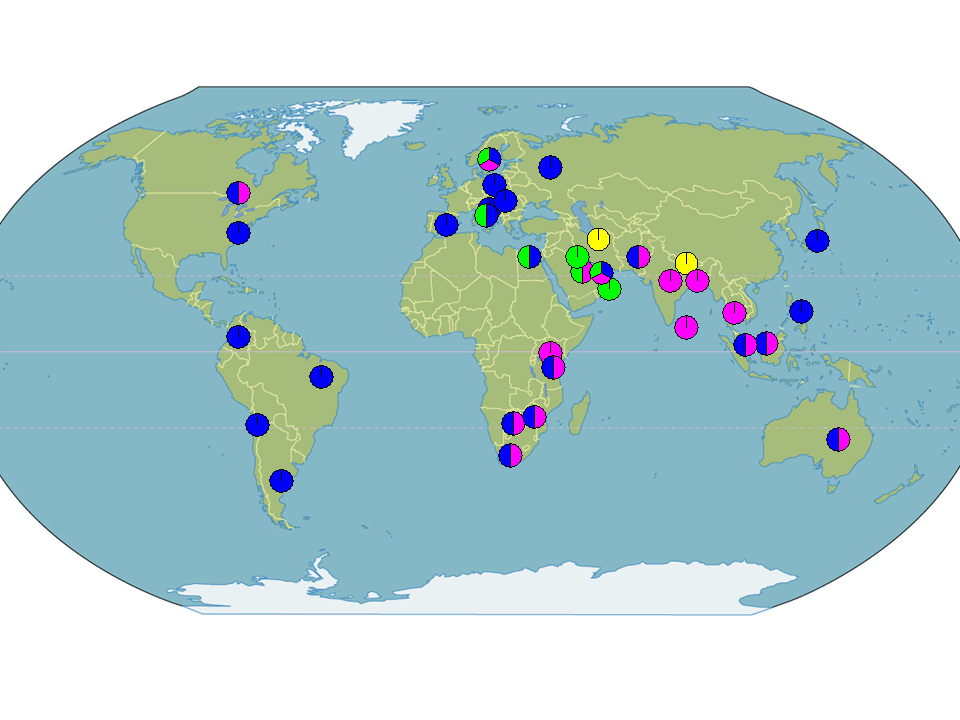

Supplement: Figure S1 — Map showing the geographic origin of each INTERHEART individual analyzed in this study. Each pie graph shows if at least one individual with self-reported ethnicity defined as “European” (blue section), “South-Asian” (pink section) or “Arabs” (green section) has been recruited in the country (regardless of the number of individuals recruited, see Supplemental Table S1 for details). All individuals from Nepal and Iran reported their ethnicity as “Other Asian” and are displayed by a yellow section. (0.50 MB TIF) [file pone.0001382.s001.tif]

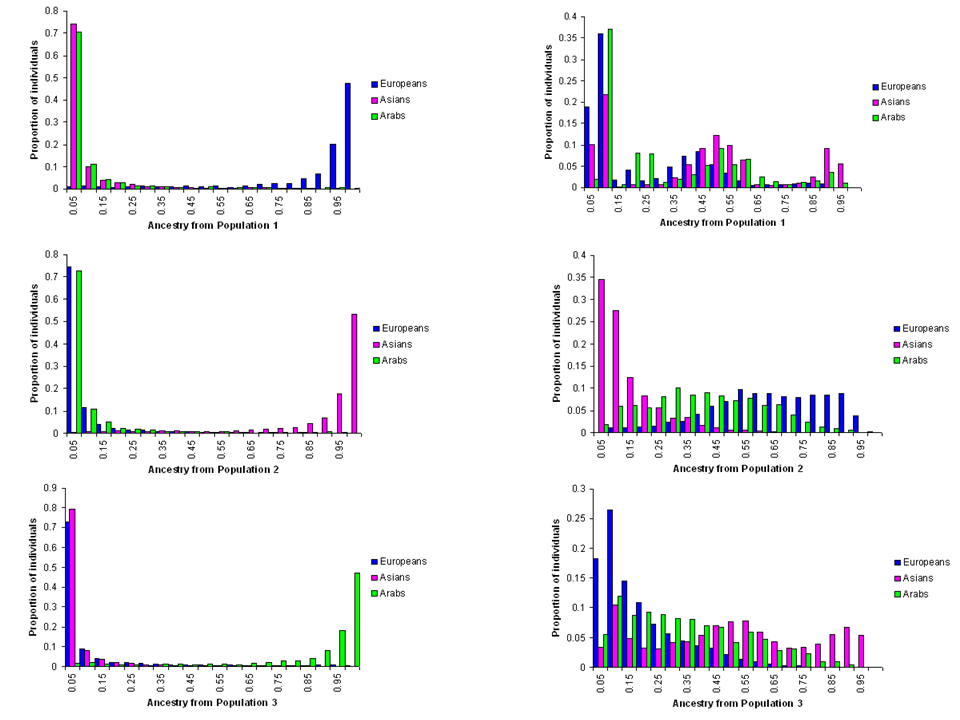

Supplement: Figure S2 — The graphs show the distribution of individuals according to their coefficients of ancestry from each population (K = 3). The left panel correspond to the assignments using 127 SNPs highly differentiated across population, the right panel to the assignments using 133 SNPs randomly selected. (0.20 MB TIF) [file pone.0001382.s002.tif]

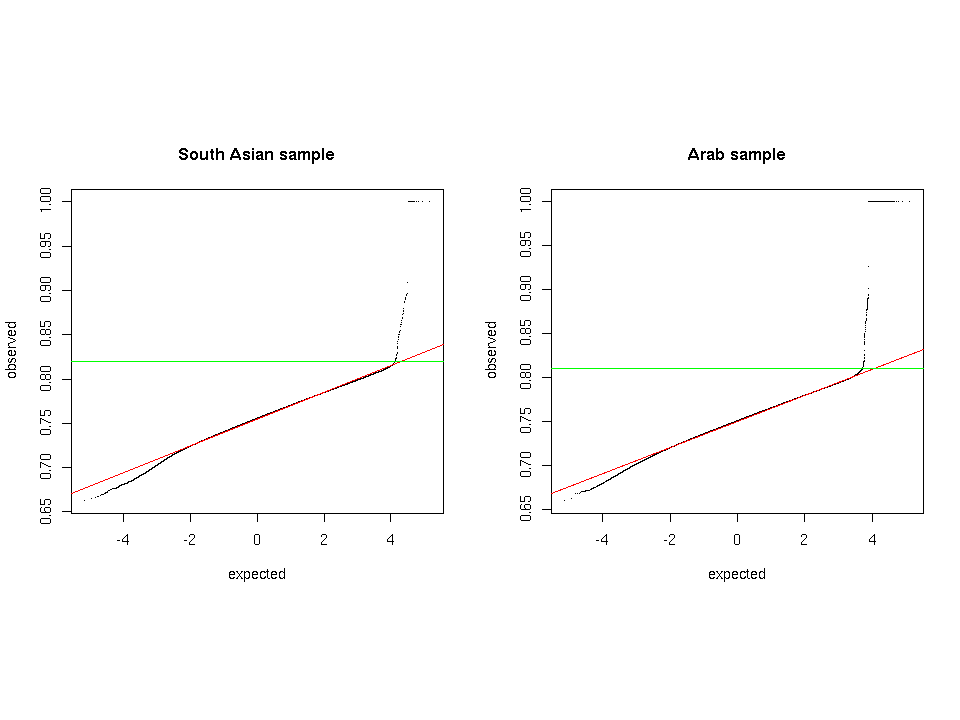

Supplement: Figure S3 — QQ plot of the distribution of pair-wise allele sharing among the South Asian (left panel) and Arab (right panel) individuals against a normal distribution. (0.06 MB TIF) [file pone.0001382.s003.tif]

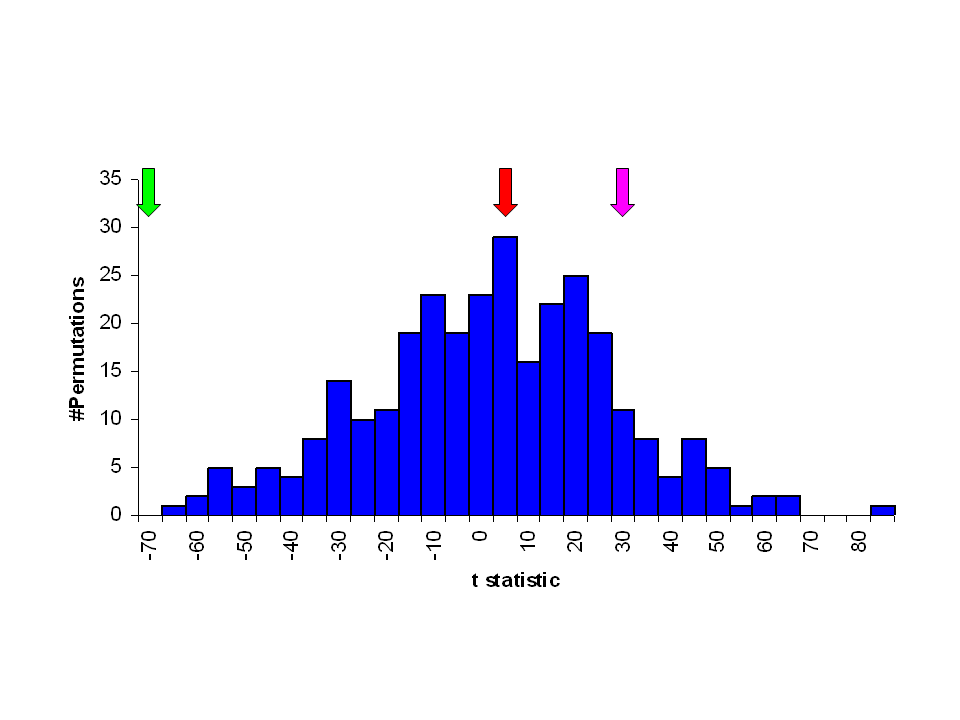

Supplement: Figure S4 — Estimation of cryptic relatedness in Europeans. The graph displays the distribution of the t-statistic obtained in 300 tests of the difference in means between the distributions of allele sharing within two groups of randomly assigned individuals (Welch Two Sample t-test). The red arrow shows the t-statistic obtained by testing the INTERHEART Europeans cases vs. controls. The green arrow corresponds to the comparison of the distribution of pair-wise allele sharing among the Saguenay Lac St-Jean (SLSJ) individuals vs. the allele sharing observed in Europeans from the INTERHEART study. The pink arrow shows the t-statistic obtained in the comparison of inter-sample allele sharing (i.e., one SLSJ individual compared to one European individual from INTERHEART) vs. the distribution of allele sharing in Europeans. (0.07 MB TIF) [file pone.0001382.s004.tif]

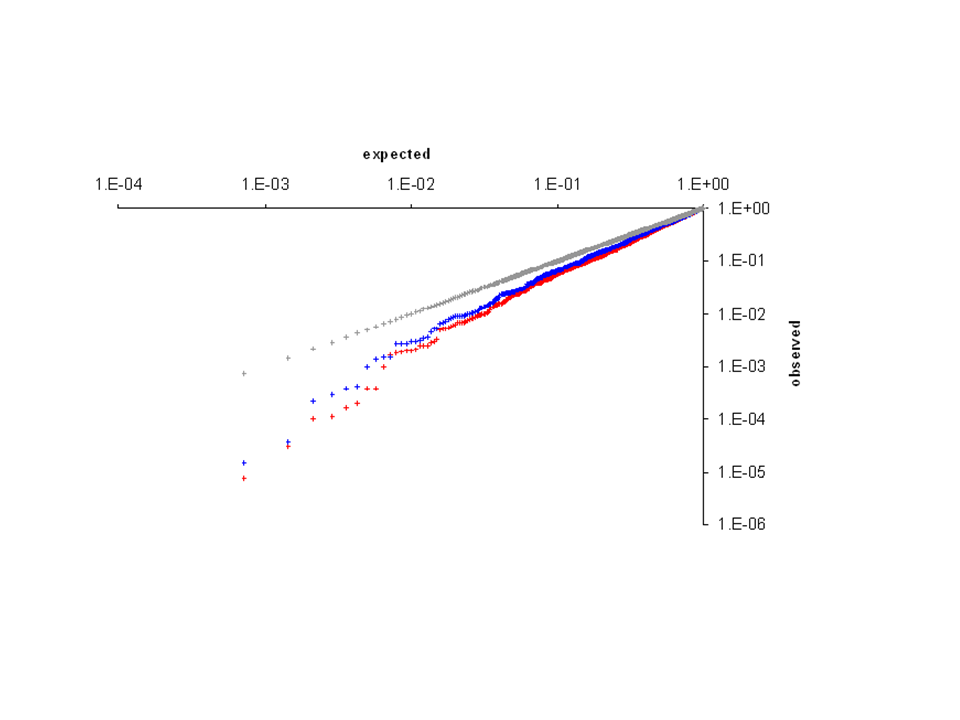

Supplement: Figure S5 — Effect of STRUCTURE on the distribution of the p-values for the associations between the genotypes and ApoB level in South-Asians. The plot shows the observed distribution of the p-values against the expectation under a model without any association (axes in logarithmic scales). Red crosses correspond to the association between ApoB and the genotypes at one SNP without any correction. Light blue crosses stand for the same tests using the coefficients of ancestry from STRUCTURE used as additional covariates (0.08 MB TIF) [file pone.0001382.s005.tif]

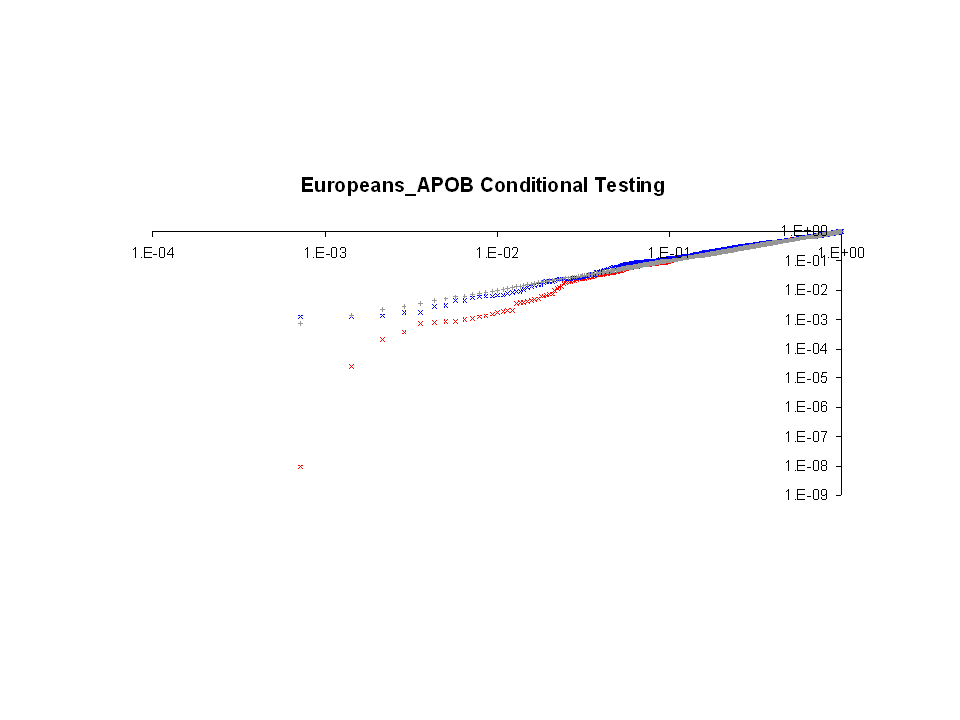

Supplement: Figure S6 — Distribution of the p-values for the associations between the genotypes and ApoB level in Europeans. Red crosses correspond to the non-corrected association between ApoB and the genotypes at one SNP. Blue crosses stands for the same tests after correcting for the signal of the five strongest associations (i.e. by conditioning the analyses on the genotypes at the five strongest associations). (0.05 MB TIF) [file pone.0001382.s006.tif]

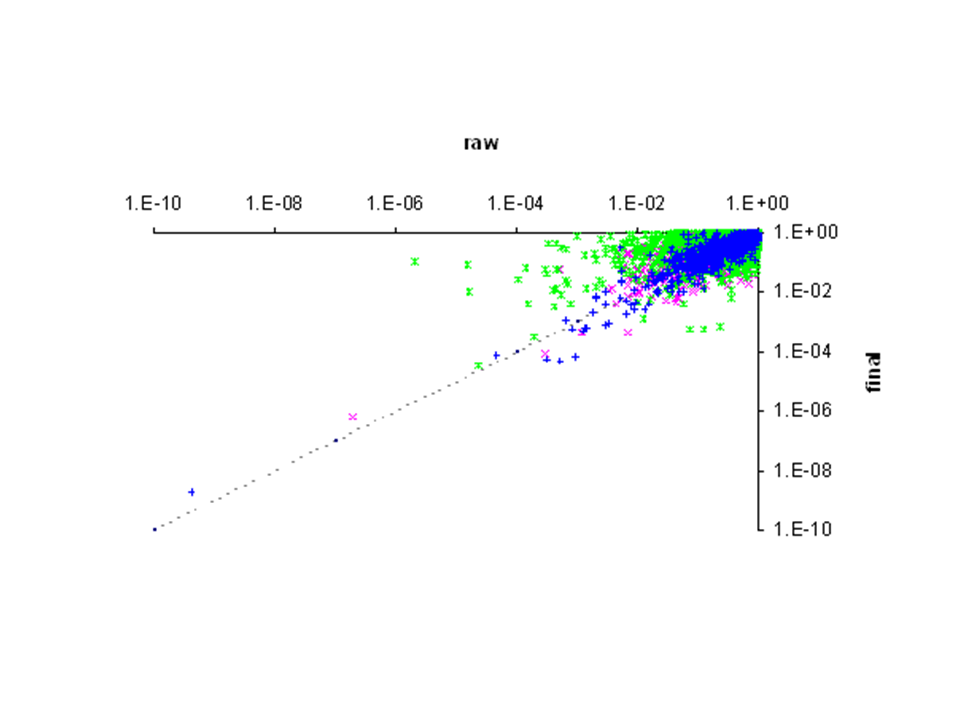

Supplement: Figure S7 — Distribution of the p-values for the associations between the genotypes and ApoB level in raw and cleaned datasets. Crosses correspond to the association between ApoB and the genotypes at one SNP using the raw (x-axis) and the cleaned datasets (y-axis). Green, Pink and Blue crosses stand for respectively the tests in the Arab, South-Asian and European datasets. (0.10 MB TIF) [file pone.0001382.s007.tif]
