# Supplementary material for: Correction of Population Stratification in Large Multi-Ethnic Association Studies
Source: PLoS One. 2008 Jan 2;3(1):e1382. doi: 10.1371/journal.pone.0001382 (PMC2198793; doi:10.1371/journal.pone.0001382)
Supplement: Table S1 — Description of the SNPs included in this study. (0.04 MB PDF) [file pone.0001382.s008.pdf]

Supplemental Table 1. Description of the SNPs included in this study.

Locus	RS ID	codingSNPs	taggingSNPs	miscSNPs	Failed
OR13G1	rs1151640			1	
PALLADIN	rs12510359			1	
ROS1	rs619203			1	1
TAS2R50	rs1376251			1	
ABCA1	rs2515606		1		
ABCA1	rs2482430		1		
ABCA1	rs2482432		1		
ABCA1	rs363717		1	1	
ABCA1	rs1331924		1		
ABCA1	rs2066881		1		
ABCA1	rs2274871		1		
ABCA1	rs2740484		1		
ABCA1	rs2297405		1		
ABCA1	rs2297406		1		
ABCA1	rs2020927		1		
ABCA1	rs2297408		1		
ABCA1	rs2740481		1		
ABCA1	rs2230808	1		1	
ABCA1	rs2740479		1		
ABCA1	rs2297404		1		
ABCA1	rs1999431		1		
ABCA1	rs1883023		1		
ABCA1	rs2066716		1		
ABCA1	rs2777802		1		
ABCA1	rs2740476		1		
ABCA1	rs2297409		1		
ABCA1	rs3780540		1		
ABCA1	rs2234885		1		
ABCA1	rs2297401		1		1
ABCA1	rs3818688		1		
ABCA1	rs4149316		1		
ABCA1	rs2254884		1		
ABCA1	rs3780542		1		
ABCA1	rs4149313	1		1	
ABCA1	rs3780543		1		
ABCA1	rs2066717		1		
ABCA1	rs2515629		1		
ABCA1	rs2065412		1		
ABCA1	rs2297398		1		
ABCA1	rs2297400		1		1
ABCA1	rs2274873		1		
ABCA1	rs2472448		1		
ABCA1	rs2487054		1		
ABCA1	rs4149291		1		
ABCA1	rs4149290		1		
ABCA1	rs1175293		1		
ABCA1	rs2487037		1		
ABCA1	rs2253304		1		
ABCA1	rs2253182		1		

Locus	RS ID	codingSNPs	taggingSNPs	miscSNPs	Failed
ABCA1	rs2253172		1		
ABCA1	rs2230806	1	1	1	
ABCA1	rs2230805		1		
ABCA1	rs2249891		1		
ABCA1	rs4149281		1		
ABCA1	rs4743764		1		
ABCA1	rs1929842		1		
ABCA1	rs1929841		1		
ABCA1	rs2000069		1		
ABCA1	rs12346461		1		
ABCA1	rs1999429		1		1
ABCA1	rs4149272		1		
ABCA1	rs3858075		1		
ABCA1	rs2275542		1		
ABCA1	rs2417565		1		
ABCA1	rs3847302		1		
ABCA1	rs2275544		1		
ABCA1	rs2275545		1		1
ABCA1	rs2740494		1		
ABCA1	rs3847305		1		
ABCA1	rs7848844		1		
ABCA1	rs2740492		1		
ABCA1	rs2575875		1		
ABCA1	rs3758294		1		
ABCA1	rs2740487		1		
ABCA1	rs2777793		1		
ABCA1	rs2575876		1		1
ABCA1	rs2740486		1		
ABCA1	rs2575879			1	
ABCA1	rs10820743		1		
ABCA1	rs10521071		1		
ABCA1	rs2437818		1		
ABCA1	rs10512336		1		
ABCA1	rs2515616		1		
ABCA1	rs2791952		1		
ABCA1	rs2472510		1		
ABCA1	rs7035693		1		
ABCA1	rs2515614		1		
ABCA1	rs2487052		1		
ABCA1	rs1800977		1		
ABCA1	rs2740483			1	
ABCA1	rs2246293		1		
ABCA1	rs2422493			1	
ABCA1	rs2487042		1		
ABCA1	rs2472493		1		
ABCA1	rs2515608		1		
ABCA1	rs2472490		1		
ABCA1	rs3887137		1		
ABCG1	rs2839477		1		
ABCG1	rs221950		1		
ABCG1	rs748319		1		

Locus	RS ID	codingSNPs	taggingSNPs	miscSNPs	Failed
ABCG1	rs8131660		1		
ABCG1	rs1378577		1		
ABCG1	rs4148086		1		
ABCG1	rs1117640		1		
ABCG1	rs8129752		1		
ABCG1	rs8127716		1		
ABCG1	rs915845		1		
ABCG1	rs4148095		1		
ABCG1	rs4148101		1		
ABCG1	rs225411		1		
ABCG1	rs3827225		1		
ABCG1	rs3787970		1		
ABCG1	rs881395		1		
ABCG1	rs225440		1		
ABCG1	rs915847		1		
ABCG1	rs225444		1		
ABCG1	rs9982196		1		
ABCG1	rs4148117		1		
ABCG1	rs225371		1		
ABCG1	rs6586298		1		
ABCG1	rs225374		1		
ABCG1	rs225376		1		1
ABCG1	rs7279750		1		
ABCG1	rs225378		1		
ABCG1	rs183436		1		
ABCG1	rs915842		1		
ABCG1	rs225385		1		
ABCG1	rs225387		1		
ABCG1	rs225393		1		
ABCG1	rs225398		1		
ABCG1	rs691687		1		
ABCG1	rs7280003		1		
ABCG1	rs2234718		1		1
ABCG1	rs4148123		1		
ABCG1	rs3787995		1		
ABCG1	rs225406		1		
ABCG1	rs4148125		1		
ABCG1	rs2839482		1		
ABCG1	rs225410		1		
ABCG1	rs492338		1		
ABCG1	rs3788006		1		
ABCG1	rs3788007		1		
ABCG1	rs425215		1		
ABCG1	rs914189		1		
ABCG1	rs2298690		1		
ABCG1	rs2276235		1		
ABCG1	rs3788010		1		
ABCG1	rs1541290		1		
ABCG1	rs2839483		1		
ABCG1	rs2839485		1		
ABCG1	rs7283700		1		

Locus	RS ID	codingSNPs	taggingSNPs	miscSNPs	Failed
ABCG1	rs15661		1		
ABCG1	rs7276176		1		
ABCG1	rs8128478		1		
ABCG1	rs225435		1		
ABCG4	rs3809046		1		1
ABCG4	rs668033		1		
ABCG4	rs626776		1		
ABCG4	rs3802885		1		
ACDC	rs1648707		1		
ACDC	rs822387		1		
ACDC	rs266729		1		
ACDC	rs182052		1		
ACDC	rs822395			1	
ACDC	rs822396		1	1	
ACDC	rs9877202		1		
ACDC	rs2241766			1	
ACDC	rs1501299			1	
ACDC	rs1063537		1		
ACDC	rs1063539		1		
ACDC	rs1403697		1		
ACDC	rs6444175		1		
ACDC	rs7628649		1		
ACE	rs4459609		1		
ACE	rs9912458		1		
ACE	rs1800764			1	
ACE	rs4291			1	
ACE	rs4295		1		
ACE	rs4303		1		
ACE	rs4305		1		
ACE	rs4309		1		
ACE	rs4311		1		
ACE	rs4316		1		
ACE	rs4318	1	1		1
ACE	rs4343			1	
ACE	rs4976	1			
ACE	rs4362		1		
ACE	rs4364	1	1		
ACE	rs4461142		1		
ACE	rs4459610	1	1		
ACE	rs8066276		1		
ACE	rs12451328		1		
ACE	rs4968591		1		
ACE	rs9914151		1		
ACE	rs9898552		1		
ADH1c	rs2066702			1	
ADH1c	rs1229984			1	
ADH1c	rs2866152		1		
ADH1c	rs1229863		1		
ADH1c	rs1614972		1		
ADH1c	rs698			1	
ADH1c	rs904096		1		

Locus	RS ID	codingSNPs	taggingSNPs	miscSNPs	Failed
ADH1c	rs2241894		1		
ADH1c	rs1662037		1		
AGT	rs3789662		1		
AGT	rs7536290		1		
AGT	rs943580		1		
AGT	rs3789670		1		
AGT	rs3789671		1		
AGT	rs2478545		1		
AGT	rs699	1		1	
AGT	rs4762	1	1		
AGT	rs5039	1			
AGT	rs2148582		1		
AGT	rs5051			1	1
AGT	rs2071405			1	
AGT	rs2071404			1	
AGT	rs7549009		1		
AGT	rs1326886		1		
AGTR1	rs422858		1		
AGTR1	rs2638363		1		
AGTR1	rs2131127		1		
AGTR1	rs931490		1		
AGTR1	rs4681443		1		
AGTR1	rs718858		1		
AGTR1	rs3772616		1		
AGTR1	rs7427804		1		
AGTR1	rs385338		1		
AGTR1	rs275649		1		
AGTR1	rs3772608		1		
AGTR1	rs6801836		1		
AGTR1	rs2320019		1		
AGTR1	rs275646		1		
AGTR1	rs275645		1		
AGTR1	rs427832		1		
AGTR1	rs2675513		1		
ALDH2	rs737280		1		
ALDH2	rs7974339		1		
ALDH2	rs7978737		1		1
ALDH2	rs886205		1		
ALDH2	rs4767944		1		
ALDH2	rs2238151		1		
ALDH2	rs671	1	1	1	
ALDH2	rs11066034		1		
ALOX5	rs1864414		1		
ALOX5	rs3780894		1		
ALOX5	rs2228064		1		
ALOX5	rs745986		1		
ALOX5	rs11239505		1		
ALOX5	rs2029253		1		
ALOX5	rs7099684		1		
ALOX5	rs7917687		1		1
ALOX5	rs1369214		1		

Locus	RS ID	codingSNPs	taggingSNPs	miscSNPs	Failed
ALOX5	rs2115819		1		
ALOX5	rs10900213		1		
ALOX5	rs10900215		1		
ALOX5	rs11239524		1		
ALOX5	rs892691		1		
ALOX5	rs3780901		1		
ALOX5	rs3780906		1		
ALOX5	rs3740107		1		
ALOX5	rs1487562		1		
ALOX5	rs7099874		1		
ALOX5	rs1059696		1		
ALOX5	rs2291427		1		
ALOX5	rs2288619		1		
ALOX5AP	rs4238134		1		
ALOX5AP	rs4076128		1		
ALOX5AP	rs3809376		1		
ALOX5AP	rs4769055		1		
ALOX5AP	rs10507391		1		
ALOX5AP	rs4769873		1		
ALOX5AP	rs3885907		1		
ALOX5AP	rs3803277		1		
ALOX5AP	rs4769874			1	
ALOX5AP	rs9579648		1		
ALOX5AP	rs4468448		1		
ALOX5AP	rs9551963			1	
ALOX5AP	rs4769058		1		
ALOX5AP	rs12019512		1		
ALOX5AP	rs9508835		1		
ALOX5AP	rs4445746		1		
ALOX5AP	rs4491352		1		
ALOX5AP	rs4769062		1		
APOA1BP	rs6682716		1		
APOA1BP	rs6700828		1		
APOA1BP	rs4661189		1		
APOA1BP	rs879461		1		
APOA1BP	rs3748574		1		
APOA1BP	rs4661193		1		1
APOA1BP	rs12023410		1		
APOA2	rs10797094		1		
APOA2	rs11587213		1		
APOA2	rs11421		1		
APOA2	rs7528588		1		
APOA2	rs3813627		1		
APOA2	rs4073054		1		
APOA2	rs2307424		1		
APOA4_APOC3_APOA1	rs675			1	
APOA4_APOC3_APOA1	rs5108			1	
APOA4_APOC3_APOA1	rs5101			1	
APOA4_APOC3_APOA1	rs5092			1	
APOA4_APOC3_APOA1	rs2542051			1	
APOA4_APOC3_APOA1	rs2854116			1	

Locus	RS ID	codingSNPs	taggingSNPs	miscSNPs	Failed
APOA4_APOC3_APOA1	rs2070668	1			
APOA4_APOC3_APOA1	rs4520	1			
APOA4_APOC3_APOA1	rs5128			1	
APOA4_APOC3_APOA1	rs5081			1	
APOA4_APOC3_APOA1	rs5077	1		1	1
APOA4_APOC3_APOA1	rs5074	1			
APOA4_APOC3_APOA1	rs12718465	1			
APOA4_APOC3_APOA1	rs5070			1	
APOA4_APOC3_APOA1	rs5069	1		1	
APOA5	rs618923		1		
APOA5	rs12285095		1		
APOA5	rs2266788			1	1
APOA5	rs619054			1	
APOA5	rs2075291			1	
APOA5	rs3135507			1	
APOA5	rs2072560			1	
APOA5	rs2542058			1	
APOA5	rs3135506			1	
APOA5	rs651821			1	
APOA5	rs648450			1	
APOA5	rs662799		1	1	
APOA5	rs1729410		1		
APOA5	rs11216137		1		
APOA5	rs6589568		1		
APOB	rs4371387		1		
APOB	rs1042034	1		1	
APOB	rs1801702	1			
APOB	rs1042031	1	1	1	
APOB	rs1800479			1	
APOB	rs1801701	1	1	1	
APOB	rs2163204		1		
APOB	rs676210	1	1	1	
APOB	rs693		1	1	
APOB	rs533617	1			
APOB	rs673548		1	1	
APOB	rs3791981		1		
APOB	rs12714192	1			
APOB	rs679899	1		1	
APOB	rs520354		1		
APOB	rs550619		1		
APOB	rs6752026	1			
APOB	rs1367117	1		1	
APOB	rs934197			1	
APOB	rs512535		1	1	
APOB	rs1713223		1		
APOB	rs7575840		1		
APOC4_APOC2	rs1132899	1	1		
APOC4_APOC2	rs5167	1	1		
APOC4_APOC2	rs5168	1			
APOC4_APOC2	rs2288911		1		
APOC4_APOC2	rs5122	1			

Locus	RS ID	codingSNPs	taggingSNPs	miscSNPs	Failed
APOC4_APOC2	rs5126	1	1		
APOC4_APOC2	rs3760627		1		
APOC4_APOC2	rs2239375		1		
APOE	rs8106922		1		
APOE	rs405509		1	1	
APOE	rs440446			1	
APOE	rs429358	1		1	
APOE	rs7412	1		1	1
APOE	rs439401		1		
APOE	rs5114		1		
APOE	rs389261		1		
APOE	rs10424339		1		
APOH	rs758767		1		
APOH	rs8178952		1		
APOH	rs6933		1		
APOH	rs4581	1	1		1
APOH	rs2215415		1		
APOH	rs8178847	1	1		
APOH	rs3815410		1		
APOH	rs8178835		1		
APOH	rs3760292		1		
APOH	rs7215391		1		1
APOH	rs9903986		1		
APOM	rs2242656		1		
APOM	rs10484558		1		
APOM	rs3117582		1		
APOM	rs805264		1		
APOM	rs707921		1		
APOM	rs3130617		1		
APOM	rs7029		1		
APOM	rs805257		1		
CCR5	rs2856758			1	
CD14	rs778583		1		
CD14	rs778584		1		
CD14	rs4914		1		
CD14	rs2569193		1		
CD14	rs5744431		1		
CD14	rs3822356		1		
CD36	rs819437		1		
CD36	rs7789369		1		
CD36	rs10499856		1		
CD36	rs10499857		1		
CD36	rs1524597		1		
CD36	rs1761663			1	
CD36	rs1722505		1		
CD36	rs1984112			1	
CD36	rs4545029		1		
CD36	rs2151916		1		
CD36	rs1572230		1		
CD36	rs10215288		1		
CD36	rs1953298		1		1

Locus	RS ID	codingSNPs	taggingSNPs	miscSNPs	Failed
CD36	rs1334511		1		
CD36	rs1049654		1		1
CD36	rs1527463		1		
CD36	rs3211810		1		
CD36	rs3211816		1		
CD36	rs3211828		1		
CD36	rs3211870		1		
CD36	rs1924		1		
CD36	rs17154246		1		
CD36	rs3211938		1		
CD36	rs7755		1		
CD36	rs1049673			1	
CD36	rs13230419		1		
CD36	rs17154304		1		
CD36	rs17266080		1		
CETP	rs3764261		1		
CETP	rs4783961			1	
CETP	rs1800775			1	1
CETP	rs820299		1		
CETP	rs1532625			1	
CETP	rs1532624		1		
CETP	rs4784744		1		
CETP	rs5880	1	1	1	
CETP	rs5882	1	1	1	
CETP	rs1800777			1	
CETP	rs289741		1		
CETP	rs289747		1		
CETP	rs1566439		1		
CRP	rs2808630		1	1	
CRP	rs3093069		1		
CRP	rs1205		1	1	
CRP	rs1130864		1		
CRP	rs1800947		1	1	
CRP	rs1417938			1	
CRP	rs3091244			1	1
CRP	rs3093058			1	
CYBA	rs899729		1		
CYBA	rs2254073		1		
CYBA	rs4782390		1		
CYBA	rs4673	1	1		1
CYBA	rs3794624		1		1
CYBA	rs9932581			1	1
CYBA	rs8854		1		
CYBA	rs3736112		1		
CYBA	rs3736110		1		
CYP1A1	rs4986880		1		
CYP1A1	rs1048943			1	
CYP1A1	rs4646421		1		
CYP1A1	rs3809585		1		
CYP1A1	rs2470893		1		
CYP1A1	rs4886605		1		

Locus	RS ID	codingSNPs	taggingSNPs	miscSNPs	Failed
CYP1B1	rs163078		1		
CYP1B1	rs2256327		1		
CYP1B1	rs9309020		1		
CYP1B1	rs10916		1		
CYP1B1	rs1800440	1	1		
CYP1B1	rs1056836	1	1	1	
CYP1B1	rs162561		1		
CYP1B1	rs2551188		1		
CYP1B1	rs162556		1		
CYP1B1	rs10175338		1		
CYP3A5	rs6956305		1		
CYP3A5	rs15524		1		
CYP3A5	rs10256106		1		
CYP3A5	rs4646450		1		
CYP3A5	rs3800959		1		
CYP3A5	rs776746		1		
CYP3A5	rs4646446		1		
EDN1	rs9471392		1		
EDN1	rs9369217		1		
EDN1	rs9380973		1		
EDN1	rs3087459		1		
EDN1	rs1630736		1		
EDN1	rs5370	1	1		
EDN1	rs2859338		1		
EDN1	rs7776339		1		
EDN1	rs7356986		1		
EDN1	rs6906760		1		
EDN1	rs6458188		1		
EDN2	rs13376620		1		
EDN2	rs12069358		1		
EDN2	rs734359		1		
EDN2	rs11572364		1		
EDN2	rs2759257		1		
EDN2	rs3754287		1		
EDN2	rs6671507		1		
EPHX1	rs868966		1		
EPHX1	rs2854450		1		
EPHX1	rs3753658		1		
EPHX1	rs1877724		1		
EPHX1	rs2671272		1		
EPHX1	rs1051740	1	1	1	
EPHX1	rs2260863		1		
EPHX1	rs2740170		1		
EPHX1	rs2234922	1	1	1	1
EPHX1	rs1051741		1		
EPHX1	rs3753663		1		
EPHX1	rs360063		1		
EPHX1	rs1009668		1		
ESR1	rs2881766		1		
ESR1	rs488133		1		
ESR1	rs532010		1		

Locus	RS ID	codingSNPs	taggingSNPs	miscSNPs	Failed
ESR1	rs6920483		1		
ESR1	rs10484922		1		
ESR1	rs7766762		1		1
ESR1	rs3853248		1		
ESR1	rs11155813		1		
ESR1	rs11155814		1		
ESR1	rs827423		1		
ESR1	rs3853251		1		
ESR1	rs2234693		1		
ESR1	rs9340820		1		
ESR1	rs827419		1		
ESR1	rs712221		1		
ESR1	rs1709183		1		1
ESR1	rs9340835		1		
ESR1	rs6557170		1		
ESR1	rs1913474		1		
ESR1	rs7761846		1		
ESR1	rs2347867		1		
ESR1	rs4870061		1		
ESR1	rs4870062		1		
ESR1	rs988328		1		
ESR1	rs9397456		1		
ESR1	rs12154178		1		
ESR1	rs1801132		1		
ESR1	rs3020314		1		
ESR1	rs3020317		1		
ESR1	rs1884051		1		
ESR1	rs985694		1		
ESR1	rs1884052		1		
ESR1	rs1884054		1		
ESR1	rs10484862		1		
ESR1	rs7764144		1		
ESR1	rs726281		1		
ESR1	rs932477		1		
ESR1	rs3020407		1		
ESR1	rs7754762		1		
ESR1	rs7757956		1		
ESR1	rs9340949		1		
ESR1	rs722208		1		
ESR1	rs2207230		1		
ESR1	rs2207231		1		
ESR1	rs2207232		1		
ESR1	rs9478265		1		
ESR1	rs926779		1		
ESR1	rs3020364		1		
ESR1	rs3020368		1		
ESR1	rs1884151		1		
ESR1	rs3778082		1		
ESR1	rs3778089		1		
ESR1	rs3798575		1		
ESR1	rs2982896		1		

Locus	RS ID	codingSNPs	taggingSNPs	miscSNPs	Failed
ESR1	rs3778090		1		
ESR1	rs3020383		1		
ESR1	rs3778099		1		
ESR1	rs3798757		1		
ESR1	rs2813544		1		
ESR1	rs2747649		1		
ESR1	rs1543403		1		
ESR1	rs2813545		1		
ESR1	rs910416		1		
ESR2	rs1152579		1		
ESR2	rs1256063		1		
ESR2	rs1256061		1		
ESR2	rs10144225		1		
ESR2	rs8017441		1		
ESR2	rs1256049		1		
ESR2	rs7154455		1		
ESR2	rs1256030		1		
ESR2	rs6573553		1		
ESR2	rs7159462		1		
ESR2	rs1952586		1		
ESR2	rs1887994		1		
ESR2	rs1271572		1		
ESR2	rs3020450		1		
FABP1	rs1530272		1		
FABP1	rs2970892		1		
FABP1	rs1441644		1		
FABP1	rs2197076		1		
FABP1	rs2241883	1	1		
FABP1	rs1530273		1		
FABP1	rs2970901		1		
FABP1	rs2860391		1		
FABP1	rs7581571		1		
FABP1	rs4386315		1		
FABP2	rs1397607		1		
FABP2	rs10518301		1		
FABP2	rs1546502		1		
FABP2	rs878372		1		
FABP2	rs1799883	1			
FABP2	rs1397613		1		
FDFT1_CTSB	rs2645444		1		
FDFT1_CTSB	rs10503424		1		
FDFT1_CTSB	rs2686187		1		
FDFT1_CTSB	rs9644754		1		
FDFT1_CTSB	rs1047643		1		
FDFT1_CTSB	rs9650662		1		
FDFT1_CTSB	rs10098874		1		
FDFT1_CTSB	rs2132709		1		
FDFT1_CTSB	rs1616534		1		
FDFT1_CTSB	rs1534863		1		
FDFT1_CTSB	rs7013950		1		
FDFT1_CTSB	rs7015547		1		

Locus	RS ID	codingSNPs	taggingSNPs	miscSNPs	Failed
FDFT1_CTSB	rs904011		1		
FDFT1_CTSB	rs2645424		1		
FDFT1_CTSB	rs1298295		1		
FDFT1_CTSB	rs11250168		1		1
FDFT1_CTSB	rs3258		1		
FDFT1_CTSB	rs2446		1		
FDFT1_CTSB	rs1296028		1		
FDFT1_CTSB	rs1692821		1		
FDFT1_CTSB	rs6731		1		
FDFT1_CTSB	rs1736078		1		
FDFT1_CTSB	rs709822		1		
FDFT1_CTSB	rs17573	1			
FDFT1_CTSB	rs1736090		1		
FDFT1_CTSB	rs1803250	1			
FDFT1_CTSB	rs1293291		1		
FDFT1_CTSB	rs1122182		1		
FDFT1_CTSB	rs6980952		1		
FDFT1_CTSB	rs1293288		1		
FDFT1_CTSB	rs1293309		1		
FDFT1_CTSB	rs1692804		1		
FGB_FGA_FGG	rs12642469		1		
FGB_FGA_FGG	rs7673587		1		
FGB_FGA_FGG	rs1800790			1	
FGB_FGA_FGG	rs4220	1	1		
FGB_FGA_FGG	rs4463047		1		
FGB_FGA_FGG	rs6825454		1		
FGB_FGA_FGG	rs4308349		1		
FGB_FGA_FGG	rs2070022		1		
FGB_FGA_FGG	rs6050	1	1		
FGB_FGA_FGG	rs2070016		1		
FGB_FGA_FGG	rs2070023		1		
FGB_FGA_FGG	rs7659613		1		
FGB_FGA_FGG	rs11930511		1		
FGB_FGA_FGG	rs2066861		1		
FGB_FGA_FGG	rs2066860		1		
FGB_FGA_FGG	rs6063	1			
FGB_FGA_FGG	rs2066870		1		
FGB_FGA_FGG	rs1800792		1		
FGB_FGA_FGG	rs7681423		1		
HMGCR_COL4A3BP	rs3931914		1		
HMGCR_COL4A3BP	rs3843481		1		
HMGCR_COL4A3BP	rs3761738		1		
HMGCR_COL4A3BP	rs12916		1		1
HMGCR_COL4A3BP	rs4703670		1		
HMGCR_COL4A3BP	rs3733907		1		
HMGCR_COL4A3BP	rs698912		1		
HMGCR_COL4A3BP	rs3761743		1		
HMGCR_COL4A3BP	rs3827608		1		
HMGCR_COL4A3BP	rs6453133		1		
HMGCR_COL4A3BP	rs7722940		1		
HMGCR_COL4A3BP	rs3889900		1		

Locus	RS ID	codingSNPs	taggingSNPs	miscSNPs	Failed
HMGCR_COL4A3BP	rs4385188		1		
HMGCR_COL4A3BP	rs6872314		1		
HMGCR_COL4A3BP	rs10070207		1		
HMGCR_COL4A3BP	rs4440336		1		
HMGCR_COL4A3BP	rs7718634		1		
HMGCR_COL4A3BP	rs12519897		1		
HMGCR_COL4A3BP	rs6891560		1		
HMGCR_COL4A3BP	rs4549504		1		
HMGCR_COL4A3BP	rs5744545		1		
HMOX1	rs735266		1		
HMOX1	rs5995096		1		
HMOX1	rs2071746		1		
HMOX1	rs6518952		1		
HMOX1	rs2071749		1		
HMOX1	rs5755720		1		
HMOX1	rs743811		1		
HMOX1	rs1078979		1		
HMOX1	rs743813		1		
HMOX1	rs4645741		1		
HMOX1	rs4645742		1		
HMOX1	rs737777		1		
HMOX1	rs713618		1		1
HMOX1	rs713873		1		
HTR1A	rs1364043		1		
HTR1A	rs1423691		1		
HTR1A	rs878567		1		
HTR1A	rs7448024		1		
IL-4	rs2243204		1		
IL-4	rs2243300		1		
IL-4	rs762534		1		
IL-4	rs734244		1		1
IL-4	rs2243266		1		
IL-4	rs2243289		1		
IL-4	rs2243291		1		
IL-4	rs11242122		1		
IL-6	rs1546762		1		
IL-6	rs1880242		1		
IL-6	rs10499563		1		
IL-6	rs2056576		1		
IL-6	rs3087221		1		
IL-6	rs2069824		1		
IL-6	rs1800795			1	
IL-6	rs2069830		1		
IL-6	rs2069832		1		
IL-6	rs1548216		1		
IL-6	rs2069843		1		
IL-6	rs11766273		1		
INSIG1	rs9690040		1		
INSIG1	rs9767875		1		1
INSIG1	rs9768687		1		
INSIG1	rs9769826		1		1

Locus	RS ID	codingSNPs	taggingSNPs	miscSNPs	Failed
INSIG1	rs9770068		1		1
INSIG1	rs9769506		1		
INSIG1	rs12381375		1		
INSIG2	rs2422166		1		
INSIG2	rs2161830		1		
INSIG2	rs1352083		1		
INSIG2	rs2161829		1		
INSIG2	rs10490625		1		
INSIG2	rs889904		1		
INSIG2	rs10490624		1		
INSIG2	rs12623648		1		
INSIG2	rs3849327		1		
INSIG2	rs2113485		1		
ITGB3	rs2015049		1		
ITGB3	rs2056131		1		
ITGB3	rs4525555		1		
ITGB3	rs3892084		1		
ITGB3	rs10514919		1		
ITGB3	rs8074094		1		
ITGB3	rs3851806		1		
ITGB3	rs2015729		1		
ITGB3	rs1000232		1		
ITGB3	rs5918	1	1		1
ITGB3	rs5919		1		
ITGB3	rs951351		1		
ITGB3	rs2292864		1		
ITGB3	rs999323		1		
ITGB3	rs3760372		1		
ITGB3	rs3809863		1		
ITGB3	rs3809865		1		
ITGB3	rs6504833		1		
KL	rs211247		1		
KL	rs570919		1		
KL	rs398655		1		
KL	rs2040414		1		
KL	rs495392		1		
KL	rs2283368		1		
KL	rs211239		1		
KL	rs526906		1		
KL	rs564823		1		
KL	rs480780		1		
KL	rs577912		1		
KL	rs7982726		1		
KL	rs685417		1		
KL	rs1888057		1		
KL	rs657049		1		
KL	rs7986435		1		
KL	rs9527025	1		1	
KL	rs522796		1		
KL	rs648202		1		
KL	rs677332		1		

Locus	RS ID	codingSNPs	taggingSNPs	miscSNPs	Failed
KL	rs582524		1		
KL	rs537008		1		
LCAT	rs2301246		1		
LCAT	rs1076887		1		
LCAT	rs11075663		1		1
LCAT	rs20549		1		1
LCAT	rs5923		1	1	1
LCAT	rs4986970	1	1	1	
LCAT	rs1109166		1	1	
LCAT	rs2292318		1		
LDLR	rs7249753		1		
LDLR	rs11672123		1		1
LDLR	rs6511720			1	
LDLR	rs8104576		1		
LDLR	rs8102912			1	
LDLR	rs6511721			1	
LDLR	rs2228671		1	1	
LDLR	rs2304183		1		
LDLR	rs10423288		1		
LDLR	rs11669576		1		
LDLR	rs10402435		1		
LDLR	rs5930		1	1	
LDLR	rs688		1		
LDLR	rs5925			1	
LDLR	rs5927		1	1	1
LDLR	rs2738459			1	
LDLR	rs2569538		1		
LDLR	rs2304182		1		
LDLR	rs13306506			1	
LDLR	rs1433099		1	1	
LDLRAP1	rs4075184		1		1
LDLRAP1	rs11563		1		
LDLRAP1	rs4659356		1		
LEP	rs791608		1		
LEP	rs10249476		1		
LEP	rs10487506		1		
LEP	rs2278815		1		
LEP	rs12706832		1		
LEP	rs7791621		1		
LEP	rs7795794		1		
LEP	rs3828942		1		
LEP	rs2060715		1		
LEPR	rs10493377		1		
LEPR	rs3806318		1		
LEPR	rs1327118		1		
LEPR	rs9436299		1		
LEPR	rs3790433		1		
LEPR	rs7883		1		
LEPR	rs11804091		1		
LEPR	rs970468		1		
LEPR	rs970467		1		

Locus	RS ID	codingSNPs	taggingSNPs	miscSNPs	Failed
LEPR	rs9436747		1		
LEPR	rs4655811		1		
LEPR	rs2025804		1		
LEPR	rs2025805		1		
LEPR	rs10158579		1		
LEPR	rs1327121		1		
LEPR	rs1327120		1		
LEPR	rs1327116		1		
LEPR	rs1171276		1		
LEPR	rs1171279		1		
LEPR	rs1022981		1		
LEPR	rs1782763		1		
LEPR	rs1409802		1		
LEPR	rs6673324		1		
LEPR	rs1137100	1	1		
LEPR	rs3790428		1		
LEPR	rs3790426		1		
LEPR	rs1343981		1		
LEPR	rs10493379		1		
LEPR	rs10493380		1		
LEPR	rs3828039		1		
LEPR	rs2154380		1		
LEPR	rs1137101	1			
LEPR	rs4655537		1		
LEPR	rs2376018		1		
LEPR	rs4567312		1		
LEPR	rs1892535		1		
LEPR	rs1805096		1		
LGALS2	rs2235335		1		
LGALS2	rs5995463		1		
LGALS2	rs2076087		1		
LGALS2	rs2281098		1		
LGALS2	rs2235338		1		
LGALS2	rs2281097		1		
LGALS2	rs4821669		1		1
LGALS2	rs7291467		1	1	
LGALS2	rs5750457		1		
LGALS2	rs140059		1		
LGALS2	rs5995472		1		
LGALS2	rs7290515		1		
LGALS2	rs9622650		1		
LIPC	rs417344		1		
LIPC	rs4774297		1		
LIPC	rs1800588		1		
LIPC	rs8192701		1		1
LIPC	rs8034802		1		
LIPC	rs6494006		1		
LIPC	rs261341		1		
LIPC	rs483140		1		
LIPC	rs261336		1		
LIPC	rs3825776		1		

Locus	RS ID	codingSNPs	taggingSNPs	miscSNPs	Failed
LIPC	rs4775048		1		
LIPC	rs10518976		1		
LIPC	rs936960		1		
LIPC	rs10518978		1		
LIPC	rs7169744		1		
LIPC	rs12148268		1		
LIPC	rs1968685		1		
LIPC	rs4775053		1		
LIPC	rs12441205		1		
LIPC	rs4775058		1		
LIPC	rs1869137		1		
LIPC	rs1869138		1		
LIPC	rs12593880		1		
LIPC	rs4561401		1		
LIPC	rs1869139		1		
LIPC	rs1973028		1		
LIPC	rs1869144		1		
LIPC	rs16940391		1		
LIPC	rs10518982		1		
LIPC	rs2899631		1		
LIPC	rs4774302		1		
LIPC	rs1979178		1		
LIPC	rs2899632		1		
LIPC	rs8028759		1		
LIPC	rs10518983		1		
LIPC	rs9652472		1		
LIPC	rs1968687		1		
LIPC	rs4775072		1		
LIPC	rs4775073		1		
LIPC	rs10518984		1		
LIPC	rs1971544		1		
LIPC	rs6078	1			
LIPC	rs690		1		
LIPC	rs11852861		1		
LIPC	rs6083	1	1		
LIPC	rs2242064		1		
LIPC	rs1869146		1		
LIPC	rs7178362		1		
LIPC	rs2414597		1		
LIPC	rs3829462		1		
LIPC	rs3829460		1		
LIPC	rs6074		1		
LIPC	rs4774308		1		
LIPE	rs10415999		1		
LIPE	rs2229614		1		1
LIPE	rs1903724		1		
LIPE	rs7248439		1		
LIPE	rs10422283		1		1
LIPE	rs11671846		1		
LIPG	rs12962061		1		
LIPG	rs3813082		1		

Locus	RS ID	codingSNPs	taggingSNPs	miscSNPs	Failed
LIPG	rs874565		1		
LIPG	rs2000812		1		
LIPG	rs2000813	1		1	
LIPG	rs2097055		1		
LIPG	rs8093249		1		
LIPG	rs11875600		1		
LIPG	rs2276269		1		
LIPG	rs12970066		1		1
LIPG	rs2276270		1		
LIPG	rs11873722		1		
LIPG	rs6507931		1		
LIPG	rs9952025		1		
LIPG	rs3744843		1		1
LIPG	rs3786248		1		
LIPG	rs9962414		1		
LIPG	rs11664186		1		
LIPG	rs4939585		1		
LIPG	rs8099385		1		
LPA	rs3124785		1		
LPA	rs7449650		1		
LPA	rs9457933		1		
LPA	rs6919346		1		
LPA	rs3798220		1		
LPA	rs11751605		1		
LPA	rs1801693		1		1
LPA	rs7761293		1		
LPA	rs9364559		1		
LPA	rs6923877		1		
LPA	rs3798221		1		
LPA	rs7765781		1		
LPA	rs6922216		1		
LPA	rs7759633		1		
LPA	rs1569933		1		
LPA	rs1406889		1		
LPA	rs1321196		1		
LPA	rs1321195		1		
LPA	rs9346833		1		
LPA	rs783149		1		
LPA	rs1406888		1		
LPL	rs7009128		1		
LPL	rs1534649		1		
LPL	rs6997330		1		
LPL	rs3779788			1	
LPL	rs1121923		1		
LPL	rs248		1		
LPL	rs255		1	1	
LPL	rs256			1	
LPL	rs263		1		
LPL	rs264		1		
LPL	rs271			1	
LPL	rs285			1	

Locus	RS ID	codingSNPs	taggingSNPs	miscSNPs	Failed
LPL	rs295		1		
LPL	rs316		1		
LPL	rs328	1		1	
LPL	rs10099160		1		
LPL	rs1059611		1		
LPL	rs10100638		1		
LPL	rs10092029		1		
LPL	rs2898493		1		
LPL	rs10096633		1		
LPL	rs1441777		1		
LTA_TNF	rs3131637		1		1
LTA_TNF	rs2844482		1		
LTA_TNF	rs2857713		1		
LTA_TNF	rs1041981	1	1	1	
LTA_TNF	rs769177		1		
LTA_TNF	rs3093559		1		
MMP13	rs7115014		1		
MMP13	rs11824755		1		
MMP13	rs3819089		1		
MMP13	rs640198		1		
MMP13	rs597315		1		
MMP13	rs3758856		1		
MMP13	rs659383		1		
MMP13	rs687558		1		
MMP3	rs473238		1		
MMP3	rs595840		1		
MMP3	rs683878		1		
MMP3	rs520540		1		
MMP3	rs3025066		1		
MMP3	rs679620	1			
MMP3	rs615098		1		
MTHFR	rs7538516		1		
MTHFR	rs6697244		1		
MTHFR	rs4846048		1		
MTHFR	rs868014		1		
MTHFR	rs2274976	1	1		
MTHFR	rs1476413		1		
MTHFR	rs1801131	1		1	
MTHFR	rs1801133	1	1		
MTHFR	rs7525338		1		
MTHFR	rs1931226		1		
MTHFR	rs3737964		1		
MTHFR	rs12404124		1		
MTP	rs1503777		1		
MTP	rs2866164			1	
MTP	rs3811800		1		
MTP	rs1800591			1	
MTP	rs6532821		1		
MTP	rs2306986	1	1		
MTP	rs3816873	1			
MTP	rs1057613		1		

Locus	RS ID	codingSNPs	taggingSNPs	miscSNPs	Failed
MTP	rs3805335		1		
MTP	rs3792683	1			
MTP	rs982424		1		
MTP	rs2306985	1			
MTP	rs745075		1		
MTP	rs1491238		1		
MTP	rs881980		1		
MTP	rs10516446		1		
MTP	rs10516448		1		
MTP	rs958868		1		
MTP	rs10516449		1		
MTP	rs7659550		1		
NAT2	rs7013253		1		
NAT2	rs4271002		1		
NAT2	rs9987109		1		
NAT2	rs1390358		1		
NAT2	rs7832071		1		
NAT2	rs1801279		1		
NAT2	rs1801280	1		1	
NAT2	rs1799930	1		1	
NAT2	rs1208	1	1	1	
NAT2	rs721398		1		
NAT2	rs1390359		1		
NAT2	rs1495737		1		
NAT2	rs2410561		1		
NAT2	rs1587145		1		
NPC1	rs1788820		1		
NPC1	rs891387		1		
NPC1	rs891386		1		
NPC1	rs1808579		1		
NPC1	rs1805084		1		
NPC1	rs10502439		1		
NPC1	rs1788823		1		
NPC1	rs6507716		1		
NPC1	rs2282558		1		
NPC1	rs1805082	1	1		
NPC1	rs3745023		1		
NPC1	rs1788799	1			
NPC1	rs2435307		1		
NPC1	rs1652343		1		
NPC1	rs1788762		1		
NPC1	rs1631685		1		
NPC1	rs1805081	1	1		
NPC1	rs2960578		1		
NPC1	rs1788783		1		
NPC1	rs8097842		1		
NPC1	rs1429934		1		
NPC1	rs2981422		1		
NPC1	rs1620047		1		
NPC1	rs1652357		1		
NPC1	rs1652358		1		

Locus	RS ID	codingSNPs	taggingSNPs	miscSNPs	Failed
NPC1	rs1617407		1		
NPC1	rs1652359		1		
NPC1L1	rs217440		1		
NPC1L1	rs217437		1		
NPC1L1	rs217434		1		
NPC1L1	rs217429		1		
NPC1L1	rs10272471		1		
NPC1L1	rs217420		1		
NPC1L1	rs2072183			1	
NPC1L1	rs10260606		1		
NPPA_NPPB	rs198408		1		
NPPA_NPPB	rs198414		1		
NPPA_NPPB	rs198358		1		1
NPPA_NPPB	rs5065	1	1		
NPPA_NPPB	rs5063	1	1		
NPPA_NPPB	rs632793		1		
NPPA_NPPB	rs198375		1		
NPPA_NPPB	rs198388		1		
NPPA_NPPB	rs5230	1			
NPPA_NPPB	rs5229	1			
NPPA_NPPB	rs5227	1	1		
NPPA_NPPB	rs1009592		1		
NQO1	rs10517		1		
NQO1	rs1800566	1	1	1	
NQO1	rs689452		1		
NQO1	rs1437135		1		
NQO1	rs689460		1		
NQO1	rs689459		1		
NQO1	rs2917666		1		
PCK1	rs1543375		1		
PCK1	rs1328757		1		
PCK1	rs707555	1	1		
PCK1	rs8192708	1	1		
PCK1	rs1804160	1			
PCK1	rs2179706		1		
PCK1	rs6025631		1		
PCK1	rs2865391		1		
PCK1	rs6128083		1		
PCTP	rs2033113		1		
PCTP	rs2912546		1		
PCTP	rs2114443		1		
PCTP	rs890487		1		1
PCTP	rs2960072		1		
PCTP	rs2332513		1		
PCTP	rs4303611		1		
PCTP	rs2960065		1		
PCTP	rs7211024		1		
PECAM1	rs1122800		1		
PECAM1	rs7214769		1		
PECAM1	rs8082170		1		
PECAM1	rs6808		1		

Locus	RS ID	codingSNPs	taggingSNPs	miscSNPs	Failed
PECAM1	rs1550792		1		
PECAM1	rs2070784		1		
PECAM1	rs4968721		1		
PECAM1	rs9913080		1		
PECAM1	rs4968723		1		
PECAM1	rs8072342		1		
PECAM1	rs7207019		1		
PECAM1	rs7218481		1		
PECAM1	rs8069023		1		
PECAM1	rs4968724		1		
PECAM1	rs2070782		1		
PECAM1	rs6504224		1		
PECAM1	rs7503550		1		
PECAM1	rs668		1		
PECAM1	rs8066263		1		
PECAM1	rs8067268		1		
PECAM1	rs8065316		1		
PLA2G7	rs9472822		1		
PLA2G7	rs974670		1		
PLA2G7	rs10498771		1		
PLA2G7	rs1051931	1	1		
PLA2G7	rs2216465		1		
PLA2G7	rs1805018	1	1		1
PLA2G7	rs1805017	1	1		
PLA2G7	rs3799863		1		
PLA2G7	rs1421368		1		
PLA2G7	rs1421378		1		
PLA2G7	rs1421379		1		
PLTP	rs742035		1		
PLTP	rs4608591		1		
PLTP	rs1736493		1		
PLTP	rs11569636		1		
PLTP	rs378114		1		1
PLTP	rs394643		1		
PLTP	rs4810479		1		
PLTP	rs3848714		1		
PON1	rs854541		1		
PON1	rs854542		1		
PON1	rs854547		1		
PON1	rs8491		1		
PON1	rs854548		1		
PON1	rs854555		1		
PON1	rs662	1	1	1	
PON1	rs2074354		1		1
PON1	rs854560	1		1	
PON1	rs3917498		1		
PON1	rs705378		1		
PON1	rs3917485		1		
PON1	rs757158		1		
PON1	rs13228784		1		
PON2	rs13226149		1		

Locus	RS ID	codingSNPs	taggingSNPs	miscSNPs	Failed
PON2	rs11977702		1		
PON2	rs6954345	1		1	
PON2	rs10487133		1		
PON2	rs987539		1		
PON2	rs11545941	1			
PON2	rs2068604		1		
PON2	rs1639		1		
PON2	rs1034809		1		1
PON2	rs2286233		1		
PON2	rs2299267		1		
PON2	rs730365		1		
PON2	rs6946850		1		
PON2	rs6978425		1		
PON2	rs43044		1		
PPARA	rs6008923		1		1
PPARA	rs135557		1		
PPARA	rs717926		1		
PPARA	rs135556		1		
PPARA	rs4253617		1		
PPARA	rs4253622		1		
PPARA	rs4253623		1		
PPARA	rs135552		1		
PPARA	rs135550		1		
PPARA	rs135549		1		
PPARA	rs135547		1		
PPARA	rs9626730		1		1
PPARA	rs4253640		1		
PPARA	rs135538		1		
PPARA	rs4253652		1		
PPARA	rs4253653		1		
PPARA	rs4253655		1		
PPARA	rs4253662		1		
PPARA	rs4253678		1		
PPARA	rs1555208		1		
PPARA	rs4253681		1		
PPARA	rs9627100		1		
PPARA	rs7364220		1		
PPARA	rs4253701		1		
PPARA	rs12330015		1		
PPARA	rs8138102		1		
PPARA	rs11703495		1		1
PPARA	rs4253712		1		1
PPARA	rs4823613		1		
PPARA	rs4253719		1		
PPARA	rs5766741		1		
PPARA	rs5766743		1		1
PPARA	rs4253727		1		
PPARA	rs4253729		1		
PPARA	rs4253747		1		
PPARA	rs1800206			1	
PPARA	rs4253753		1		

Locus	RS ID	codingSNPs	taggingSNPs	miscSNPs	Failed
PPARA	rs4253754		1		
PPARA	rs4253755		1		
PPARA	rs4253757		1		
PPARA	rs12170204		1		
PPARA	rs6007662		1		
PPARA	rs5767743		1		1
PPARA	rs4253760		1		
PPARA	rs4253763		1		
PPARA	rs11090819		1		
PPARA	rs4253776		1		
PPARA	rs4253777		1		
PPARA	rs7286168		1		
PPARA	rs9627287		1		1
PPARA	rs7289611		1		
PPARG	rs2920500		1		
PPARG	rs13077495		1		1
PPARG	rs2920502		1		1
PPARG	rs2972164		1		
PPARG	rs4684846		1		
PPARG	rs10510410		1		
PPARG	rs6805419		1		
PPARG	rs6800910		1		
PPARG	rs12636454		1		
PPARG	rs7646510		1		
PPARG	rs9814788		1		1
PPARG	rs880663		1		
PPARG	rs10510418		1		
PPARG	rs2197423		1		
PPARG	rs1801282	1	1	1	
PPARG	rs4135304		1		
PPARG	rs1899951		1		
PPARG	rs1064323		1		
PPARG	rs1373640		1		
PPARG	rs4135317		1		
PPARG	rs2972162		1		
PPARG	rs10510419		1		
PPARG	rs2938395		1		
PPARG	rs4135334		1		
PPARG	rs4135268		1		
PPARG	rs1822825		1		
PPARG	rs709149		1		
PPARG	rs4135354		1		
PPARG	rs709157		1		
PPARG	rs1175540		1		
PPARG	rs4135292		1		
PPARG	rs4135360		1		
PPARG	rs3856806		1		
PPARG	rs1152003		1		
PPARG	rs6790976		1		
PPARG	rs4498025		1		
PPARG	rs1152005		1		

Locus	RS ID	codingSNPs	taggingSNPs	miscSNPs	Failed
PPARGC1B	rs10515632		1		
PPARGC1B	rs11740140		1		
PPARGC1B	rs12659166		1		
PPARGC1B	rs1076064		1		
PPARGC1B	rs1124995		1		
PPARGC1B	rs899178		1		
PPARGC1B	rs11740247		1		
PPARGC1B	rs2012522		1		
PPARGC1B	rs11746690		1		
PPARGC1B	rs17711430		1		
PPARGC1B	rs4705374		1		
PPARGC1B	rs4705375		1		
PPARGC1B	rs10515638		1		
PPARGC1B	rs880769		1		
PPARGC1B	rs6579757		1		
PPARGC1B	rs4705378		1		
PPARGC1B	rs1422429		1		
PPARGC1B	rs10875551		1		
PPARGC1B	rs1030176		1		
PPARGC1B	rs1012543		1		
PPARGC1B	rs2052490		1		
PPARGC1B	rs17461842		1		
PPARGC1B	rs759814		1		
PPARGC1B	rs2161257		1		
PPARGC1B	rs2010994		1		
PPARGC1B	rs251466		1		
PPARGC1B	rs251464		1		
PPARGC1B	rs32589		1		
PPARGC1B	rs741581		1		
PPARGC1B	rs2003604		1		
PPARGC1B	rs10491361		1		
PPARGC1B	rs32579		1		
PPARGC1B	rs7732671	1	1		
PPARGC1B	rs26127		1		
PPARGC1B	rs26125		1		
PPARGC1B	rs26124		1		1
PPARGC1B	rs26120		1		
PPARGC1B	rs888853		1		
PPARGC1B	rs7712296		1		
PPARGC1B	rs3733665		1		
PTGS2	rs10911902		1		
PTGS2	rs4648307		1		
PTGS2	rs4648298			1	
PTGS2	rs2206593		1		
PTGS2	rs5275			1	
PTGS2	rs5273	1			
PTGS2	rs5272	1	1		
PTGS2	rs5278		1		
PTGS2	rs5277			1	
PTGS2	rs20417			1	
PTGS2	rs10911905		1		

Locus	RS ID	codingSNPs	taggingSNPs	miscSNPs	Failed
RETN	rs1862513		1		
RETN	rs3219175		1		
RETN	rs3219177			1	
RETN	rs3745367		1	1	
RETN	rs3745368			1	
RETN	rs3745369		1		
RETN	rs1423096		1		
RETN	rs10401670		1		
SCARB1	rs838873		1		1
SCARB1	rs838878		1		
SCARB1	rs3825140		1		1
SCARB1	rs701106		1		
SCARB1	rs838891		1		
SCARB1	rs838896		1		
SCARB1	rs838862		1		
SCARB1	rs838861		1		
SCARB1	rs1672879		1		
SCARB1	rs961170		1		
SCARB1	rs961169		1		
SCARB1	rs838911		1		
SCARB1	rs989892		1		
SCARB1	rs865716		1		
SCARB1	rs5892		1		
SCARB1	rs838900		1		
SCARB1	rs4765614		1		
SCARB1	rs5889		1		
SCARB1	rs2278986		1		
SCARB1	rs1902569		1		
SCARB1	rs12231837		1		
SCARB1	rs10846744		1		
SCARB1	rs4765616		1		
SCARB1	rs10846748		1		
SCARB1	rs4765623		1		
SCARB1	rs10773109		1		
SCARB1	rs3924313		1		
SCARB1	rs10773111		1		
SCARB1	rs10773112		1		
SCARB1	rs4765181		1		
SCARB1	rs11615630		1		
SCARB1	rs4379922		1		
SCARB1	rs10846760		1		
SCARB1	rs6488950		1		
SCARB1	rs4765633		1		
SELE	rs12043338		1		
SELE	rs4656698		1		
SELE	rs4786		1		
SELE	rs5355	1		1	
SELE	rs5368	1			
SELE	rs5366	1	1		1
SELE	rs5364	1			
SELE	rs3917419		1		

Locus	RS ID	codingSNPs	taggingSNPs	miscSNPs	Failed
SELE	rs5361	1	1	1	
SELE	rs5360	1			
SELE	rs3917403		1		
SELE	rs932307		1		
SELE	rs1805193			1	
SELE	rs7515714		1		
SELE	rs10489181		1		
SELE	rs6427213		1		
SERPINE1	rs6950982		1		
SERPINE1	rs6465787			1	
SERPINE1	rs2227631		1	1	
SERPINE1	rs6092	1		1	
SERPINE1	rs2227648		1		
SERPINE1	rs7242		1		
SERPINE1	rs2227714		1		
SLC6A4	rs7224199		1		
SLC6A4	rs1042173		1		
SLC6A4	rs2054848		1		
SLC6A4	rs3794808		1		
SLC6A4	rs140701		1		
SLC6A4	rs4583306		1		
SLC6A4	rs140700		1		
SLC6A4	rs2228673	1			1
SLC6A4	rs2020942		1		
SLC6A4	rs6355	1			
SLC6A4	rs16965628		1		
SLC6A4	rs2020933		1		
SLC6A4	rs9903062		1		
SREBF1	rs3818717		1		
SREBF1	rs4925114		1		1
SREBF1	rs2297508		1		
SREBF1	rs2282180		1		
SREBF1	rs4925115		1		
SREBF1	rs8066560		1		
SREBF1	rs9902941		1		
SREBF1	rs1889018		1		
SREBF1	rs4925118		1		1
SREBF1	rs2236513		1		
SREBF1	rs3744115		1		
SREBF2	rs5758487		1		1
SREBF2	rs1569451		1		
SREBF2	rs2006638		1		
SREBF2	rs1009544		1		
SREBF2	rs2255957		1		
SREBF2	rs133280		1		
SREBF2	rs9607852		1		
SREBF2	rs2269657		1		
SREBF2	rs11702960		1		
SREBF2	rs133291		1		
SREBF2	rs4822062		1		
SREBF2	rs4822063		1		

Locus	RS ID	codingSNPs	taggingSNPs	miscSNPs	Failed
SREBF2	rs2229440		1		
SREBF2	rs2269658		1		
SREBF2	rs1052717		1		
SREBF2	rs6002521		1		
SREBF2	rs2267443		1		
SREBF2	rs2269662		1		1
SREBF2	rs2229442		1		
THBD	rs3746726		1		
THBD	rs2567608		1		1
THBD	rs6076013		1		
THBD	rs2007363		1		
THBD	rs3176123		1		
THBD	rs1042579	1		1	
THBD	rs13306848			1	
THBD	rs6048519		1		
THBD	rs2424505		1		
THBS1	rs2618164		1		
THBS1	rs10520136		1		
THBS1	rs1478604		1		
THBS1	rs2236741		1		
THBS1	rs2292305	1	1		
THBS1	rs2292304		1		
THBS1	rs2228262			1	
THBS1	rs2228263		1		
THBS1	rs1051442		1		
THBS1	rs949646		1		1
THBS1	rs8032035		1		
THBS2	rs9505932		1		1
THBS2	rs7764005		1		
THBS2	rs10945400		1		
THBS2	rs8089		1		
THBS2	rs10945405		1		
THBS2	rs11966235		1		
THBS2	rs6940420		1		
THBS2	rs9283850		1		
THBS2	rs9379341		1		
THBS2	rs9505897		1		
THBS2	rs9765848		1		
THBS2	rs11758161		1		
THBS2	rs6940801		1		
THBS2	rs6605524		1		1
THBS2	rs9717830		1		
THBS4	rs2434273		1		
THBS4	rs3813667		1		
THBS4	rs2438650		1		
THBS4	rs404375		1		
THBS4	rs368936		1		
THBS4	rs401302		1		
THBS4	rs1866389	1	1	1	
THBS4	rs2241824		1		1
THBS4	rs4703797		1		

Locus	RS ID	codingSNPs	taggingSNPs	miscSNPs	Failed
THBS4	rs256438		1		
THBS4	rs2288394		1		
THBS4	rs10514175		1		
THBS4	rs2247450		1		
THBS4	rs7714280		1		
THBS4	rs2118732		1		
THBS4	rs2438642		1		
VCAM1	rs2050471		1		
VCAM1	rs4520436		1		
VCAM1	rs1041163		1		
VCAM1	rs3170794		1		
VCAM1	rs3176860		1		
VCAM1	rs3917010		1		
VCAM1	rs3917040		1		
VCAM1	rs3783613	1	1		
VCAM1	rs3181088		1		
VCAM1	rs3176877		1		
VCAM1	rs3176878		1		
VCAM1	rs3783615	1	1		
VCAM1	rs10493936		1		
VLDLR	rs4741745		1		
VLDLR	rs1869589		1		
VLDLR	rs1454626		1		1
VLDLR	rs2242103		1		
VLDLR	rs1545567		1		
VLDLR	rs4741747		1		
VLDLR	rs1454627		1		
VLDLR	rs1551411		1		
VLDLR	rs1869592		1		
VLDLR	rs10812381		1		
VLDLR	rs6149		1		
VLDLR	rs2242104		1		
VLDLR	rs3780181		1		
VLDLR	rs2290465		1		
VLDLR	rs6475888		1		
VLDLR	rs6148		1		
VLDLR	rs7043836		1		
VLDLR	rs3421		1		
VLDLR	rs8210		1	1	
VLDLR	rs7040916		1		
VLDLR	rs4741753		1		
VLDLR	rs1877480		1		
VLDLR	rs1947447		1		
VLDLR	rs1567712		1		
VLDLR	rs1869593		1		
VLDLR	rs2054073		1		
IL18	rs543810		1		
IL18	rs5744260		1		
IL18	rs360722		1		
IL18	rs795467		1		
IL18	rs2043055		1		

Locus	RS ID	codingSNPs	taggingSNPs	miscSNPs	Failed
IL18	rs1946518		1		
IL18	rs5744222		1		
IL18	rs360712		1		
NR1H2	rs1274513		1		
NR1H2	rs2248949		1		1
NR1H2	rs3219281		1		
NR1H2	rs3219306		1		
NR1H2	rs2546550		1		
