# Supplementary material for: Correction of Population Stratification in Large Multi-Ethnic Association Studies
Source: PLoS One. 2008 Jan 2;3(1):e1382. doi: 10.1371/journal.pone.0001382 (PMC2198793; doi:10.1371/journal.pone.0001382)
Supplement: Table S2 — Excluded samples (0.01 MB PDF) [file pone.0001382.s009.pdf]

Supplemental Table T2. Excluded samples

	European	Arab*	South Asian	Iran	Nepal*	Total*
**Genotyped**	4292	2099	2584	460	316	9751
<95% Gtyped	56	48	25	6	0	135
Sex discordance	57	122 (19)	23	4	46 (1)	252 (104)
Identical	29	109 (27)	8	8	16 (2)	170 (74)
Related	37	41 (40)	27	14	12 (10)	131 (128)
Ethnicity	44	9	51	na	na	104
Probl. Center^	na	557	na	na	162	719
**2nd Generation Dataset**	4069	1399	2450	excluded	excluded	7918

* The number in parentheses indicates the number of individuals excluded after removal of the problematic centers.
^ Includes individuals with sex discordance and identical/related individuals
