# Supplementary material for: Correction of Population Stratification in Large Multi-Ethnic Association Studies
Source: PLoS One. 2008 Jan 2;3(1):e1382. doi: 10.1371/journal.pone.0001382 (PMC2198793; doi:10.1371/journal.pone.0001382)
Supplement: Table S3 — Outliers identified by STRUCTURE with substantial ancestry from South-East Asia or Sub-Saharan Africa (0.01 MB PDF) [file pone.0001382.s010.pdf]

Supplemental Table T3. Outliers identified by STRUCTURE with substantial ancestry from South-East Asia or Sub-Saharan Africa

South-East Asia

Pop. Sample	Anc. Coeff.	Country
SAsian	0.995	Malaysia
SAsian	0.994	Qatar
SAsian	0.994	Nepal
SAsian	0.993	Thailand
SAsian	0.993	Nepal
SAsian	0.988	Nepal
SAsian	0.988	Nepal
SAsian	0.988	Malaysia
SAsian	0.988	Malaysia
SAsian	0.987	Nepal
SAsian	0.984	Nepal
SAsian	0.983	Nepal
SAsian	0.981	Nepal
SAsian	0.981	Nepal
SAsian	0.981	Nepal
SAsian	0.981	Malaysia
SAsian	0.98	Nepal
SAsian	0.979	Nepal
SAsian	0.978	Nepal
SAsian	0.977	Nepal
SAsian	0.977	Nepal
SAsian	0.976	Nepal
SAsian	0.976	Nepal
SAsian	0.976	Malaysia
SAsian	0.975	Nepal
SAsian	0.975	Nepal
SAsian	0.975	Nepal
SAsian	0.972	Nepal
SAsian	0.972	Bangladesh
SAsian	0.97	Nepal
SAsian	0.968	Nepal
SAsian	0.968	Singapore
SAsian	0.966	Nepal
SAsian	0.96	Nepal
SAsian	0.959	Nepal
SAsian	0.951	Nepal
SAsian	0.949	Nepal
SAsian	0.948	Nepal
SAsian	0.942	Nepal
SAsian	0.939	Nepal
SAsian	0.934	Nepal
SAsian	0.93	Nepal
SAsian	0.927	Nepal
SAsian	0.922	Nepal
SAsian	0.92	Nepal
SAsian	0.914	Nepal
SAsian	0.913	Nepal

Sub-Saharan Africa

Pop. Sample	Anc. Coeff.	Country
SAsian	0.993	Zimbabwe
SAsian	0.991	South Africa
SAsian	0.984	Zimbabwe
SAsian	0.633	Pakistan
SAsian	0.56	Pakistan
SAsian	0.516	Bahrain
SAsian	0.414	Mozambique
SAsian	0.377	Pakistan
SAsian	0.301	Pakistan
Arab	0.988	Oman
Arab	0.961	Oman
Arab	0.95	Oman
Arab	0.946	Quatar
Arab	0.933	Oman
Arab	0.878	Oman
Arab	0.825	Scandinavia
Arab	0.807	Oman
Arab	0.797	UAE
European	0.994	Zimbabwe
European	0.948	Zimbabwe
European	0.784	USA
European	0.754	Zimbabwe
European	0.662	Brazil
European	0.645	Brazil
European	0.517	Brazil
European	0.495	Brazil
European	0.484	SouthAfrica
European	0.465	SouthAfrica
European	0.427	UAE
European	0.386	Brazil
European	0.354	Brazil
European	0.309	Australia
European	0.294	Brazil
European	0.287	Australia
European	0.24	Australia
European	0.239	Brazil
European	0.233	Scandinavia
European	0.225	Brazil
European	0.225	UAE
European	0.221	Egypt

SAsian	0.911	Nepal
SAsian	0.909	Nepal
SAsian	0.896	Bangladesh
SAsian	0.893	Nepal
SAsian	0.893	Bangladesh
SAsian	0.887	India
SAsian	0.878	Nepal
SAsian	0.873	Nepal
SAsian	0.868	Nepal
SAsian	0.867	Nepal
SAsian	0.862	Nepal
SAsian	0.855	Nepal
SAsian	0.854	Nepal
SAsian	0.839	UAE
SAsian	0.839	Nepal
SAsian	0.836	Nepal
SAsian	0.828	Nepal
SAsian	0.826	Nepal
SAsian	0.825	Nepal
SAsian	0.823	Nepal
SAsian	0.821	Qatar
SAsian	0.815	Nepal
SAsian	0.805	Nepal
SAsian	0.805	Nepal
SAsian	0.793	Nepal
SAsian	0.789	Nepal
SAsian	0.787	Nepal
SAsian	0.782	Qatar
SAsian	0.782	Nepal
SAsian	0.781	Nepal
SAsian	0.775	Nepal
SAsian	0.769	Nepal
SAsian	0.766	Bangladesh
SAsian	0.765	Bangladesh
SAsian	0.731	Nepal
SAsian	0.726	India
SAsian	0.712	Nepal
SAsian	0.709	Qatar
SAsian	0.707	Nepal
SAsian	0.703	Nepal
SAsian	0.701	Nepal
SAsian	0.701	Bangladesh
SAsian	0.7	Bangladesh
SAsian	0.699	Nepal
SAsian	0.692	Nepal
SAsian	0.691	Nepal
SAsian	0.682	Nepal
SAsian	0.679	Pakistan
SAsian	0.677	Bangladesh
SAsian	0.676	Nepal
SAsian	0.675	Pakistan
SAsian	0.669	Nepal

SAsian	0.668	Nepal
SAsian	0.668	Nepal
SAsian	0.667	Bangladesh
SAsian	0.664	Bangladesh
SAsian	0.644	Bangladesh
SAsian	0.637	Malaysia
SAsian	0.636	Nepal
SAsian	0.636	Nepal
SAsian	0.627	Nepal
SAsian	0.625	Bangladesh
SAsian	0.617	Nepal
SAsian	0.613	Nepal
SAsian	0.613	Nepal
SAsian	0.61	Nepal
SAsian	0.601	Nepal
SAsian	0.6	Pakistan
SAsian	0.6	Singapore
SAsian	0.592	Nepal
SAsian	0.588	Nepal
SAsian	0.585	Bangladesh
SAsian	0.567	Bangladesh
SAsian	0.559	Nepal
SAsian	0.558	Nepal
SAsian	0.555	Pakistan
SAsian	0.551	Nepal
SAsian	0.549	Pakistan
SAsian	0.547	Bangladesh
SAsian	0.543	Nepal
SAsian	0.542	Nepal
SAsian	0.535	Bangladesh
SAsian	0.532	Pakistan
SAsian	0.53	Bangladesh
SAsian	0.523	Nepal
SAsian	0.522	UAE
SAsian	0.52	Nepal
SAsian	0.519	Bangladesh
SAsian	0.51	Nepal
SAsian	0.508	Sri Lanka
European	0.994	Japan
European	0.993	Japan
European	0.977	Philippines
European	0.708	Philippines
European	0.534	Russia
European	0.476	UAE
European	0.457	Poland
European	0.444	Poland
European	0.432	Russia
European	0.403	Russia
European	0.378	Brazil
European	0.377	Russia
European	0.37	Poland
European	0.341	Croatia

European	0.334	Italy
European	0.33	Poland
European	0.329	Croatia
European	0.324	Poland
European	0.324	Poland
European	0.316	Russia
European	0.315	Poland
European	0.306	Poland
