# Supplementary material for: Correction of Population Stratification in Large Multi-Ethnic Association Studies
Source: PLoS One. 2008 Jan 2;3(1):e1382. doi: 10.1371/journal.pone.0001382 (PMC2198793; doi:10.1371/journal.pone.0001382)
Supplement: Text S1 — Tagging Efficiency (0.42 MB DOC) [file pone.0001382.s011.doc]

**Supplemental Information: Tagging Strategy and Efficiency**

***Method***


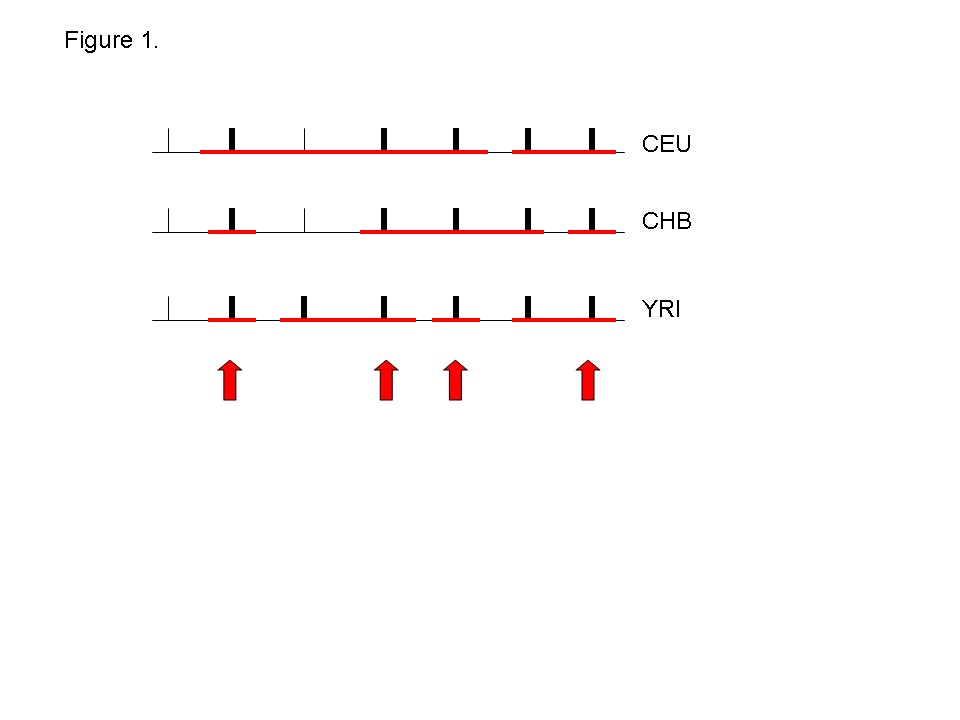
For each of the three HapMap populations used, we retrieved individual genotypes at all SNPs. We then estimated linkage disequilibrium among SNPs with a MAF larger than 5% (represented by thick vertical bars in the Figure 1) and defined “bins” of SNPs highly correlated with each others that can be tagged by a single marker (r^2^>0.8, red horizontal bars). Finally, we selected the minimal set of tSNPs (red arrows) that allows capturing most of the genetic diversity in every population.

**Figure 1.** Tagging strategy.

After identifying and removing identical and related individuals (N=301) as well as individuals with a discordance between their self-reported ethnicity and the ancestry inferred genetically (N=104), we analyzed each INTERHEART population sample separately with LD-select. We first used only genotypes from the SNPs included as tagging SNPs and the same criteria as described above (MAF>5% and r^2^>0.8). Second, we added the genotypes of the remaining SNPs (i.e., cSNPs and functional SNPs determined from the literature) and estimated whether these were in LD with one of the selected tSNPs.

***Results***

Our analyses focus on individuals from three broadly defined ethnicities (i.e. European, ‘Arab and Iranian’, and ‘South Asian and Nepalese’) that were recruited from 154 centers around the world. While genetic diversity in various European populations has been extensively studied (Hinds et al. 2005; The International HapMap Consortium 2005; de Bakker et al. 2006; Montpetit et al. 2006), our study also includes individuals from populations with very scarce (if any) information on allele frequencies and linkage disequilibrium (LD) patterns. To capture most of the genetic diversity in all ethnicities (and possibly later extend this study to other ethnicities), we selected the minimal set of SNPs such that, in each of three HapMap populations (CHB, YRI and CEU), every HapMap SNP with a minor allele frequency larger than 5% is directly genotyped or in LD (r^2^>0.8) with one of the genotyped SNPs (Figure 1).

We first assessed the efficiency of this tagging strategy in the INTERHEART samples (after removing outliers, see below) by comparing it to a tagging approach based exclusively on one of the HapMap populations. We successfully genotyped the INTERHEART individuals at 1,331 SNPs previously selected as tagging (tSNPs). Of these 1,301 (97.7%) were successfully genotyped by the HapMap project in individuals with northern or western European ancestry (CEU), while the 30 remaining SNPs were genotyped in individuals with ancestry from Africa (YRI) or East-Asia (HCB or JPT) but failed in Europeans. In CEU individuals, genotyping only 779 SNPs (60%) would be sufficient to capture all genetic information (with an r^2^ of 0.8 or more) from the 1,300 SNPs genotyped by the HapMap project (Table 2). In the European individuals from the INTERHEART study, genotyping 798 SNPs (60%) would have been sufficient to recover the genetic diversity of the 1,331 tSNPs genotyped (i.e. genotyping the remaining 533 SNPs only yield mostly redundant information). On the other hand, by genotyping only tSNPs determined using the CEU individuals, we would have missed information from 70 SNPs (~5%). Most of this loss is due to incomplete genotype information in the HapMap Phase I (which is now less of a problem with the release of Phase II SNPs), as well as to minor differences in allele frequencies and LD patterns between the HapMap CEU sample and the INTERHEART Europeans. The number of tSNPs required to capture most of the information from the SNPs genotyped increases for South-Asian and Arab individuals from the INTERHEART study with respectively 822 (61%) and 958 (72%) tagging SNPs needed. Similarly, the number of SNPs that would not have been captured at an r^2^ of 0.8 using only tSNPs determined from the HapMap CEU increases to 132 (10%) and 232 (17%) “missed” SNPs in South Asian and Arab respectively. The efficiency of tagging SNPs selected from another HapMap population is displayed in Table 1.

|  | **CEU** | **HCB** | **YRI** |  |  |
| --- | --- | --- | --- | --- | --- |
|  | **# tSNPs necessary*** | | |  |  |
|  | 779 | 694 | 1068 |  |  |
|  |  |  |  |  |  |
|  | **# tSNPs missed^** | | |  | **min. # tSNPs'''** |
| **European** | 70 (5%) | 232 (17%) | 162 (12%) |  | 798 |
| **South Asian** | 132 (10%) | 216 (16%) | 157 (12%) |  | 822 |
| **Arab** | 232 (17%) | 346 (26%) | 165 (12%) |  | 958 |
| * number of tSNPs necessary to capture all common SNPs (MAF>5%, r2>0.8) | | | | | |
| ^ number of tSNP missed using one population tagging strategy | | | | | |
| '' minimal number of tSNPs necessary to capture all common SNPs in a given population sample | | | | | |

**Table 1.** Tagging efficiency

The frequency spectra obtained for each population sample is very similar to each other (see Figure 2), which illustrates our ability to capture equal proportions of common and rare SNPs in all three population samples using this tagging strategy.

**Figure 2.** Frequency spectra of the tagging SNPs in the HapMap CEU (light blue) and the three INTERHEART samples (Europeans in dark blue, South Asian in pink and Arabs in green).

For the SNPs that we selected independently of the tagging SNPs (i.e., coding non-synonymous SNPs and functional SNPs identified from the literature), we observe that 76%, 76% and 69 % of those with a MAF larger than 5% were also selected as tSNPs or are in LD (r^2^>0.8) with one SNP genotyped in, respectively, Europeans, South Asians and Arabs.

***Discussion***

Several recent studies have looked at the transferability of HapMap tagging SNPs to other human populations (Sawyer et al. 2005; Conrad et al. 2006; de Bakker et al. 2006; Gonzalez-Neira et al. 2006; Montpetit et al. 2006). While they present somewhat conflicting results, the overall finding is that selecting tagging SNPs (tSNPs) from one HapMap population captures a large fraction of the diversity in other populations: depending on the population considered and the ascertainment of the SNPs studied, between 50 to 90% of the non-tSNPs are in high LD (i.e. r^2^>0.8) with one of the tSNPs. Not surprisingly, populations more closely related to one of the HapMap populations and/or small isolated populations tend to be more efficiently covered by HapMap tSNPs. In our study, we analyzed individuals from three broadly defined ethnicities including two large groups that have not been studied previously: Arabs and South Asians. We show that an approach based on the overlap of the tSNPs of different HapMap populations leads to a greater recovery of the genetic diversity than tSNPs defined using only a single population. This is especially true in the Arab dataset for which a tagging strategy based solely on the HapMap CEU individuals would have missed 18% of the SNPs. In addition, it is worth noting than even using this tagging strategy, close to 50% of the SNPs that had not been genotyped by the HapMap Consortium (at the time of our analysis) are not in strong LD with one of our tSNPs. This observation is consistent with the numbers presented in previous studies (Conrad et al. 2006; de Bakker et al. 2006). These figures will likely be less dramatic when data from the HapMap Phase 2 is used to select tSNPs but then, more SNPs will be required as tagging. However, this clearly shows that, while tagging allows capturing a large fraction of the genetic diversity using fewer markers, it might also miss important associations (or lead to an important loss of power due to low LD between untyped causative polymorphisms and the SNPs genotyped). Different commercial “whole genome” genotyping arrays allow quick genotyping of several hundreds of thousands of SNPs and are being used to scan the entire human genome. However, one should always keep in mind that the coverage is not complete and critically depends on the population investigated (Barrett and Cardon 2006). It might thus be worthwhile to complement this approach with custom assays targeting specific candidate markers or regions (Cardon 2006; Jorgenson and Witte 2006).

**References**

Barrett JC, Cardon LR (2006) Evaluating coverage of genome-wide association studies. Nat Genet 38(6): 659-662.

Cardon LR (2006) Genetics. Delivering new disease genes. Science 314(5804): 1403-1405.

Conrad DF, Jakobsson M, Coop G, Wen X, Wall JD et al. (2006) A worldwide survey of haplotype variation and linkage disequilibrium in the human genome. Nat Genet 38(11): 1251-1260.

de Bakker PI, Burtt NP, Graham RR, Guiducci C, Yelensky R et al. (2006) Transferability of tag SNPs in genetic association studies in multiple populations. Nat Genet 38(11): 1298-1303.

Gonzalez-Neira A, Ke X, Lao O, Calafell F, Navarro A et al. (2006) The portability of tagSNPs across populations: a worldwide survey. Genome Res 16(3): 323-330.

Hinds DA, Stuve LL, Nilsen GB, Halperin E, Eskin E et al. (2005) Whole-genome patterns of common DNA variation in three human populations. Science 307(5712): 1072-1079.

Jorgenson E, Witte JS (2006) A gene-centric approach to genome-wide association studies. Nat Rev Genet 7(11): 885-891.

Montpetit A, Nelis M, Laflamme P, Magi R, Ke X et al. (2006) An evaluation of the performance of tag SNPs derived from HapMap in a Caucasian population. PLoS Genet 2(3): e27.

Sawyer SL, Mukherjee N, Pakstis AJ, Feuk L, Kidd JR et al. (2005) Linkage disequilibrium patterns vary substantially among populations. Eur J Hum Genet 13(5): 677-686.

The International HapMap Consortium (2005) A haplotype map of the human genome. Nature 437(7063): 1299-1320.
